# An RNA-dependent RNA polymerase gene in bat genomes derived from an ancient negative-strand RNA virus

**DOI:** 10.1038/srep25873

**Published:** 2016-05-13

**Authors:** Masayuki Horie, Yuki Kobayashi, Tomoyuki Honda, Kan Fujino, Takumi Akasaka, Claudia Kohl, Gudrun Wibbelt, Kristin Mühldorfer, Andreas Kurth, Marcel A. Müller, Victor M. Corman, Nadine Gillich, Yoshiyuki Suzuki, Martin Schwemmle, Keizo Tomonaga

**Affiliations:** 1 Transboundary Animal Diseases Research Center, Joint Faculty of Veterinary Medicine, Kagoshima University, Kagoshima, Japan; 2 Institute for Virology, University Medical Center Freiburg, Freiburg, Germany; 3 College of Bioresource Sciences, Nihon University, Fujisawa, Japan; 4 Department of Viral Oncology, Institute for Virus Research, Kyoto University, Kyoto, Japan; 5 Laboratory of Veterinary Microbiology II, Department of Veterinary Medicine, Azabu University, Kanagawa, Japan; 6 Laboratory Forest Ecosystem Management, Graduate School of Agriculture, Hokkaido University, Sapporo, Japan; 7 Center for Biological Safety and Special Pathogens, Robert Koch Institute, Berlin, Germany; 8 Leibniz Institute for Zoo and Wildlife research, Berlin, Germany; 9 Institute of Virology, University of Bonn Medical Centre, Bonn, Germany; 10 German Centre for Infection Research (DZIF), partner sites Bonn-Cologne, Germany; 11 Graduate School of Natural Sciences, Nagoya City University, Nagoya, Japan; 12 Department of Tumor Viruses, Graduate School of Medicine, Kyoto University, Kyoto, Japan; 13 Department of Mammalian Regulatory Network, Graduate School of Biostudies, Kyoto University, Kyoto, Japan

## Abstract

Endogenous bornavirus-like L (EBLL) elements are inheritable sequences derived from ancient bornavirus L genes that encode a viral RNA-dependent RNA polymerase (RdRp) in many eukaryotic genomes. Here, we demonstrate that bats of the genus *Eptesicus* have preserved for more than 11.8 million years an EBLL element named eEBLL-1, which has an intact open reading frame of 1,718 codons. The eEBLL-1 coding sequence revealed that functional motifs essential for mononegaviral RdRp activity are well conserved in the EBLL-1 genes. Genetic analyses showed that natural selection operated on eEBLL-1 during the evolution of *Eptesicus*. Notably, we detected efficient transcription of eEBLL-1 in tissues from *Eptesicus* bats. To the best of our knowledge, this study is the first report showing that the eukaryotic genome has gained a riboviral polymerase gene from an ancient virus that has the potential to encode a functional RdRp.

Endogenous viral elements (EVEs) are virus-derived sequences in the genomes of eukaryotes generated by the germline integration of entire viral genomes or genome fragments. The best known examples of EVEs are endogenous retroviruses (ERVs). Retrovirus genomes integrate into the host genomic DNA as part of their replication cycle, and retroviruses become endogenous when the viruses infect host germline cells. ERVs are molecular fossils of past retrovirus infection events and provide interesting insights into co-evolution between retroviruses and their hosts^1^. Interestingly, some genes that originated from ERVs have allowed the implementation of new biological host features (i.e., placentation in mammalian species), demonstrating that viral sequences can be sources of genetic novelty in eukaryotes^2^,^3^,^4^,^5^.

Bornaviruses are non-segmented, negative-strand RNA viruses belonging to the order *Mononegavirales*^6^,^7^,^8^. Although the bornavirus replication cycle lacks DNA intermediates, we and others have found that EVEs descended from bornaviruses exist in the genomes of eukaryotes; these EVEs are designated endogenous bornavirus-like (EBL) elements^9^,^10^,^11^,^12^. To date, EBLN, EBLM, EBLG and EBLL elements homologous to the N (nucleoprotein), M (matrix protein), G (envelope glycoprotein) and L (RNA-dependent RNA polymerase: RdRp) genes of bornaviruses, respectively, have been discovered. Among these elements, the EBLNs have been well studied. Notably, some of the EBLN elements retain relatively long open reading frames (ORFs) that are transcribed into RNA^10^,^13^, leading us to speculate that they play functional roles in their host cells. Indeed, it was reported that *Homo sapiens* EBLN-2 (hsEBLN-2) interacts with several cellular proteins^14^. Recently, we demonstrated that the epigenetic control of hsEBLN-1 expression affected the transcription efficiency of a neighbouring gene^15^. Additionally, we demonstrated that the expression of *Ictidomys tridecemlineatus* EBLN (itEBLN) inhibited the replication of a modern mammalian bornavirus (Borna disease virus; BDV)^13^. We also proposed that some EBLN elements in rodent and primate genomes might function as non-coding RNAs^16^. These observations strongly suggest that RNA viruses can also be a driving force of genome evolution in their hosts.

EBLL elements are widely distributed in many eukaryote species ranging from vertebrates to arthropods^12^. A recent study demonstrated that the mosquito *Aedes aegypti* has accumulated many EVEs in its genome and that an EVE homologous to the L gene of rhabdovirus in the *Mononegavirales* family has evolved under purifying selection^17^. Additionally, it was suggested that an EVE from the parvovirus replicase gene have been exapted in degu (*Octodon degus*)^18^. Because the L of bornavirus is also an enzymatic protein, the co-option of EBLL elements may provide genetic innovations in the host species. However, little analysis has been performed for EBLL elements.

In this study, we analyzed EBLL elements in mammals to gain insights into the biological roles of EBLL elements in their hosts. Surprisingly, we found that EBLL elements in bats belonging to the genus *Eptesicus* retain intact ORFs consisting of 1,718 codons that have been maintained for at least 11.8 million years (MY). Furthermore, we showed that the EBLLs evolved under purifying selection and still possess functional motifs conserved among the *Mononegavirales* RdRps. These results strongly suggest that the EBLL elements encode functional proteins that may be RdRps.

## Results

### Discovery of a large and intact ORF in an Eptesicus fuscus EBLL element

**Figure 1:**
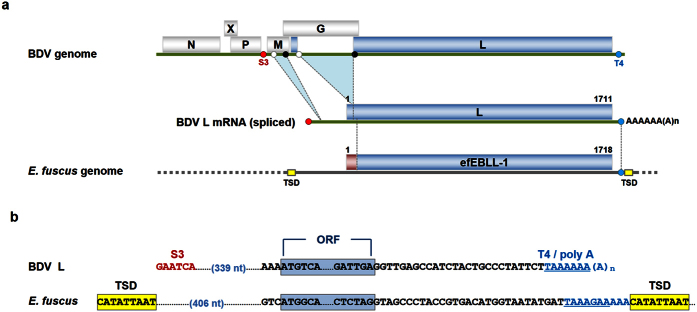
EBLL element in *Eptesicus fuscus*. (**a**) Schematic figures of the BDV genome, spliced BDV L mRNA and efEBLL-1. The numbers above the boxes indicate the positions of amino acid residues in BDV L or efEBLL-1. White and black circles represent splicing donors and acceptors in the BDV genome, respectively. Red and blue circles indicate the S3 transcription initiation and T4 transcription termination signals, respectively. Yellow boxes indicate target site duplication (TSD). The efEBLL-1 N-terminal region coloured in red does not have homology with BDV L. (**b**) Nucleotide sequence alignment of BDV L and efEBLL-1. The blue boxed areas indicate the ORFs of BDV L or efEBLL-1. Red and underlined blue letters indicate the S3 transcription start and T4 transcription termination signals (or T4-like sequences), respectively. The poly-A sequences are indicated with blue letters. Yellow boxes indicate the TSD. nt, nucleotides.

First, we conducted ORF screening for these elements in several mammalian species and found an EBLL element in the bat species *Eptesicus fuscus* (designated efEBLL-1; accession number ALEH01013293). efEBLL-1 retains a large and intact ORF of 1,718 codons that is comparable in length to the L gene of exogenous bornaviruses with 1,711 codons (Fig. 1a). A reverse pBLAST analysis revealed that the best match to efEBLL-1 was the L protein of Avian bornavirus (ABV) isolate bil (accession number EU781967; 37.1 % identity) (Supp. Table 1). Typically, the L proteins of bornaviruses are more similar to efEBLL-1 than other mononegaviral L proteins, including nyamiviruses^19^. The topology of the phylogenetic tree based on the sequences of the efEBLL-1 and L proteins of bornaviruses and nyamiviruses was consistent with the results of the reverse BLAST analysis (Supp. Fig. 1). These data suggest that efEBLL-1 is indeed derived from an ancient bornavirus.

efEBLL-1 presents a typical transcription termination signal (T4) of exogenous bornaviruses followed by a short poly-A sequence (Fig. 1b). The distance between the stop codon and the T4-like sequence of efEBLL-1 is similar to that of BDV (Fig. 1b). However, no viral transcription start signal (S3)-like sequence is present upstream of the efEBLL-1 ORF. Notably, a 9-bp direct repeat sequence exists upstream of the ORF and immediately downstream of the T4 and poly-A-like sequence (Fig. 1b); direct repeat sequences can be a landmark of a long interspersed nuclear element 1 (LINE-1)-mediated insertion (so-called target site duplication or TSD)^20^. Therefore, it is highly likely that the mRNA encoding the L gene of an ancient bornavirus integrated into the genome of the host via the LINE-1 reverse transcriptase as observed for anthropoid EBLNs^9^,^10^. The lack of an S3-like sequence upstream of the efEBLL-1 ORF may have resulted from a genomic mutation during evolution or an aberrant integration event that inserted a partial segment of the targeted mRNA, as is often observed for LINE-1-mediated integration^20^. To elucidate whether efEBLL-1 was generated from an mRNA species encoding a monocistronic L gene or polycistronic genes (i.e., M and G) (Supp. Fig. 2), we surveyed sequences homologous to several bornavirus proteins in the contig containing the efEBLL-1 locus. To this end, we performed a tBLASTn search in the whole shotgun genome sequence of *E. fuscus* using the amino acid sequences of the N, X, P, M, G or L proteins of exogenous bornaviruses. Although EBLN and EBLG elements were found in the genome of *E. fuscus* as previously reported^12^, no EBL element was found closely located to the efEBLL-1 locus, which suggests that efEBLL-1 was generated from an mRNA of transcription unit 3 (Supp. Fig. 2). We could not conclude more precisely regarding the source of the specific viral mRNA transcript from which efEBLL-1 originated due to the lack of similarity between the upstream sequence of efEBLL-1 and the genomes of modern bornaviruses (Fig. 1 and Supp. Fig. 2).

### The genus Eptesicus contains orthologous genomic efEBLL-1 sequences

To investigate whether orthologous efEBLL-1 sequences exist in the genomes of other bat species, we performed tBLASTn analysis using the deduced amino acid sequence of efEBLL-1 as a query. The tBLASTn analysis revealed homologous sequences of efEBLL-1 in bat species of the genus *Myotis* (*M. davidii* and *M. lucifugus*) (Supp. Table 2). Some of the sequences were detected in a previous report^12^. Because sequence similarities among orthologous genes in different species are usually observed in both flanking regions and coding regions, we analyzed the upstream and downstream sequences of the ORFs of the EBLLs detected by the tBLASTn analysis. However, we could not determine the gene orthology due to insertions of transposable elements and the recombination of the genomes of the genus *Myotis* (Supp. Fig. 3).

**Figure 2:**
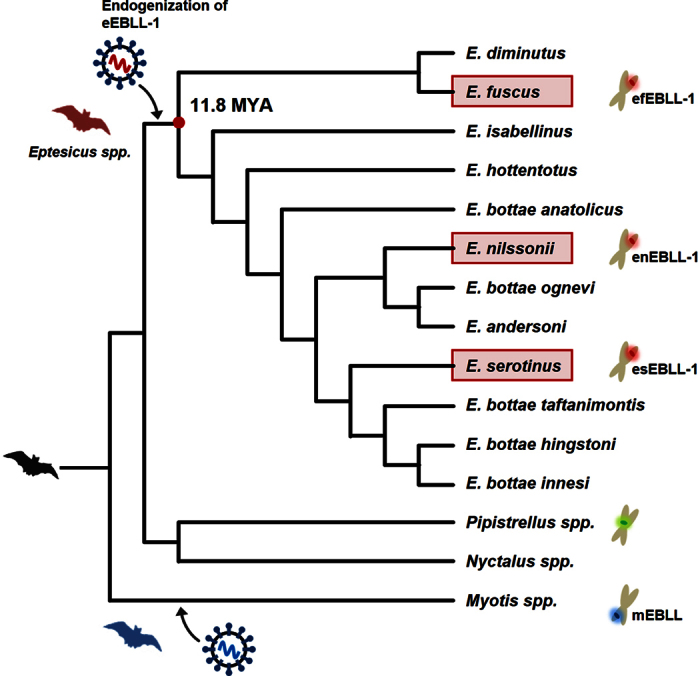
Endogenization of eEBLL-1 in the *Eptesicus* species. Schematic phylogenetic tree of the *Eptesicus* bat species. The tree topology was adopted from a previous report^47^. The arrows indicate the integration events of eEBLL-1 or EBLLs in *Myotis* bats. The red circle indicates the divergent point of the *Eptesicus* species. Red boxes show the species analyzed in this study.

Because the database search did not provide any information for the efEBLL-1 orthology, we searched for orthologous efEBLL-1 sequences in vesper bat genome sequences that were not yet deposited in the database: *E. serotinus*, *E. nilssonii*, *Pipisterillus spec*, *Nyctalus noctula* and *M. daubentonii*. We performed PCR using 3 primer sets that were designed based on the efEBLL-1 nucleotide sequence (Supp. Table 3). The expected bands amplified and sequenced from the genomes of species of the genus *Eptesicus* (*E. serotinus* and *E. nilssonii*) (Supp. Fig. 4) revealed nucleotide sequences that were almost identical to efEBLL-1 (greater than 98% identity). To determine the gene orthology, we analyzed the entire sequences of the *Eptesicus* EBLLs and their flanking regions by PCR and direct sequencing. Notably, EBLLs in *E. serotinus* and *E. nilssonii* also retained huge intact ORFs consisting of 1718 codons (accession numbers AB921210 for *E. nilssonii* and AB921214 for *E. serotinus*) with deduced amino acid sequences that were almost identical to efEBLL-1 (greater than 98 % identity). In addition, the 5′ and 3′ flanking sequences are highly conserved among efEBLL-1 and the EBLLs in *E. serotinus* and *E. nilssonii*, indicating that these EBLLs are orthologous (Supp. Fig. 5). Therefore, we designated these hypothetical genes as *Eptesicus* EBLL-1 (eEBLL-1), with the eEBLL-1 genes in *E. serotinus* and *E. nilssonii* designated esEBLL-1 and enEBLL-1, respectively. These observations suggest that the integration of an ancient bornavirus L gene occurred prior to the divergence of *E. fuscus*, *E. serotinus* and *E. nilssonii*. Based on the TimeTree^21^, the divergent time of *E. fuscus* and *E. serotinus* was 11.8 million years ago (MYA). These data suggest that the endogenization of an L sequence of an ancient bornavirus occurred at least 11.8 MYA (Fig. 2). The putative TSDs are also present in *E. serotinus* and *E. nilssonii* (Supp. Fig. 5).

### Analysis of the deduced amino acid sequence of eEBLL-1

**Figure 3:**
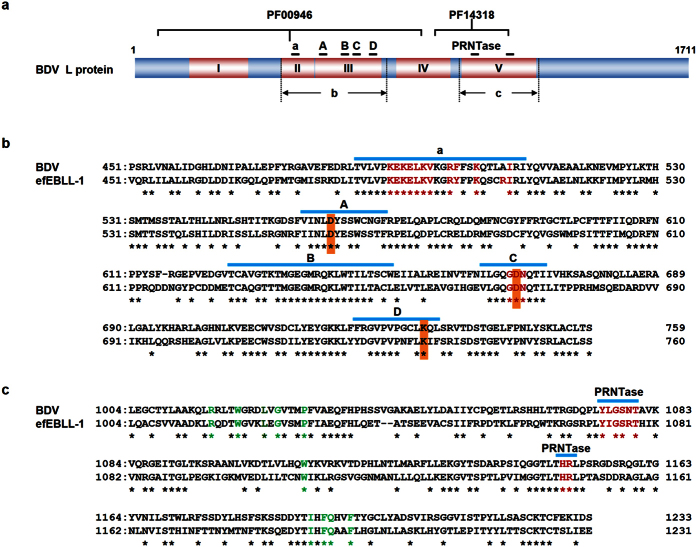
Amino acid sequence alignment between BDV L and efEBLL-1. (**a**) Schematic figure of the BDV L protein. The numbers above the box indicate the amino acid residue positions. The pink boxes show conserved blocks I-V of the mononegaviral RdRp^22^. The highly conserved stretches “a” and A–D and the PRNTase domain are shown. The regions of the possible functional domains in eEBLL-1 predicted by the Pfam search (PF00946, *Mononegavirales* RNA-dependent RNA polymerase domain and PF14318, *Mononegavirales* mRNA-capping region V domain) are shown. (**b**,**c)** Amino acid sequence alignments spanning blocks II and III (**b**) and block V (**c**) between BDV L and efEBLL-1. “a”, A, B, C and D show the conserved stretches among the mononegaviral RdRps. Orange boxes indicate strictly invariant amino acid residues among the mononegaviral RdRps. Red and green letters show functional motifs and highly conserved amino acid residues among the mononegaviral RdRps, respectively.

Next, we investigated the deduced amino acid sequence of eEBLL-1. The long-time conservation of the intact ORF in eEBLL-1 allowed us to predict that eEBLL-1 might maintain functional motifs of a viral RdRp. Therefore, we explored the functional domains in eEBLL-1 by a Pfam search. The Pfam search identified two Pfam domains [PF00946 (Mononegavirales RdRp) and PF14318 (Mononegavirales mRNA-capping region V)] in the bornavirus L and all eEBLL-1 sequences (Fig. 3a, Supp. Fig. 6a and Supp. Table 4), indicating that the eEBLL-1s and bornavirus L proteins share similar properties. We also aligned the amino acid sequences of the eEBLL-1s and L proteins of ABV and BDV (Fig. 3b,c and Supp. Fig. 6b–d). Six highly conserved blocks (block I - VI) were reported in the L proteins of mononegaviruses. The bornavirus L proteins lack block VI, which usually encodes a methyltransferase (MTase) domain^22^,^23^. Five conserved stretches (motifs a, A, B, C and D) were reported to be present in the L proteins^22^. Motif “a” exists in block II, and motifs A-D are located in block III (Fig. 3a). The amino acid sequences in the motifs “a”, A, B, C and D are well conserved among ABV L, BDV L and eEBLL-1s (Fig. 3b and Supp. Fig. 6b). Each motif was reported to contain an amino acid residue that was strictly conserved among known RNA-dependent polymerases^22^. The strictly conserved amino acid residues in mononegaviruses are also present in eEBLL-1, with the exception of motif B because bornaviruses do not contain the conserved glycine residue in motif B^6^ (Fig. 3b and Supp. Fig. 6b). The putative template-recognition site was reported to be composed of the KEKE [hydrophobic] K motif, followed by basic and hydrophobic amino acid residues spaced every four amino acids in motif “a” in block II^22^ (Fig. 3b). The bornavirus L proteins do not possess strictly conserved motif sequences, and the eEBLL-1s have stretches in the putative template-recognition sites that are similar to bornavirus L (Fig. 3b and Supp. Fig. 6b).

The GDN motif in motif B, which is essential for RdRp activity, exists in block III of the eEBLL-1s (Fig. 3b and Supp. Fig. 6b). The putative guanosine 5′-triphosphatase and RNA:GDP polyribonucleotidyltransferase (PRNTase) domain in block V, which consists of the [Y/W]xG[S/T/A]xT motif (x represents any amino acid) and the HR motif^23^,^24^, are also present in all of the eEBLL-1s (Fig. 3c and Supp. Fig. 6c). Furthermore, 10 amino acid residues in block V (green letters in Fig. 3c and Supp. Fig. 6c) that are highly conserved among mononegaviruses^24^ were also observed in the eEBLL-1s, although the function of these amino acid residues remains unclear (Fig. 3c and Supp. Fig. 6c). The lack of an MTase in the eEBLL-1s is consistent with the bornavirus L proteins. However, a putative nuclear localization signal (NLS) sequence^25^ was not found in the eEBLL-1s (Supp. Fig. 6d). Notably, the NLS sequence in BDV L is not conserved in ABV L, and a report has shown that the NLS of BDV L does not encode a functional sequence^26^.

### Detection of eEBLL-1 transcripts from Eptesicus bats

**Figure 4:**
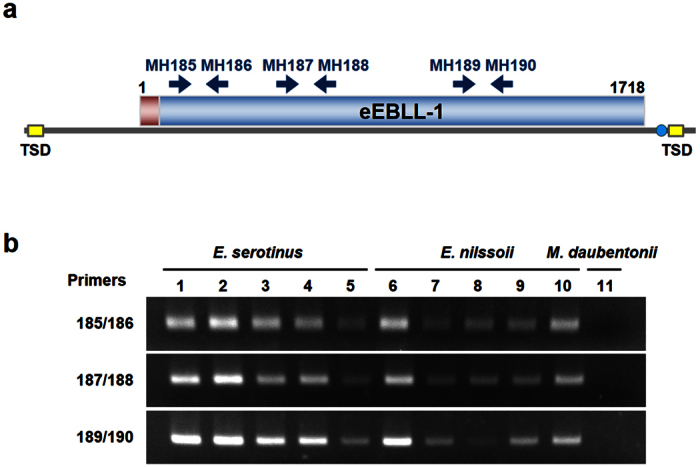
Expression of EBLL-1 RNA in the *Eptesicus* species. (**a**) Schematic figure of eEBLL-1. Primer pairs used for RT-PCR analysis are indicated. Blue circle, T4-like sequence; yellow box, target site duplication (TSD). (**b**) Detection of the expression of eEBLL-1 RNAs with different primer pairs. Primer pairs are indicated at the left. Lanes 1-5. *E. serotinus*; 6-10. *E. nilssonii*; 11. *M. daubentonii*. These gel images were cropped, and full-length gels are available in Supp. Fig. 9.

We investigated whether the eEBLL-1s are transcribed using RT-PCR with RNA samples from five individuals of *E. serotinus* and *E. nilssonii*. Notably, the expected bands were detected in the tissue samples of all ten *Eptesicus* bats but not a *Myotis* bat (Fig. 4). Therefore, the eEBLL-1s are expressed as RNAs in the *Eptesicus* species.

### Evidence for the exaptation of eEBLL-1

**Figure 5:**
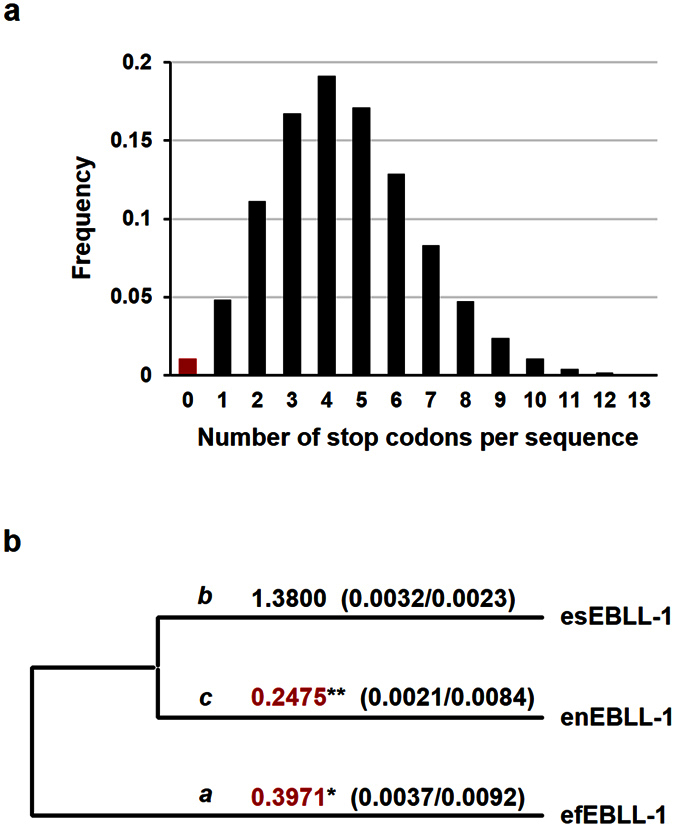
Detection of natural selection in eEBLL-1. (**a**) The distribution of the number of premature termination codons that efEBLL-1 acquired during 11.8 million years under neutral evolution from 100,000 simulation replicates. The frequency of zero stop codons is indicated with a red bar (0.0105). (**b**) The *d*_N_/*d*_S_ ratios at each branch of the eEBLL-1 phylogenetic tree. The branches where purifying selection was detected are indicated with red letters. *p < 0.05; **p < 0.01.

The maintenance of the ORFs for over 11.8 MY and the presence of the transcripts suggested that eEBLL-1 might express a functional protein in the host species. To predict the evolutionary significance of eEBLL-1s in the bats, we examined the natural selection of eEBLL-1s. First, we simulated the numbers of premature stop codons acquired by the eEBLL-1s under neutral evolution for 11.8 MY. In the simulations, the probabilities of maintaining the ORFs for 11.8 MY were significantly low (*p* = 0.01 or <0.01) (Fig. 5a, Supp. Fig. 7), which indicated that the eEBLL-1s evolved under selection pressure to keep the ORFs after endogenization.

Next, we examined natural selection at each branch of the phylogenetic tree for the eEBLL-1 ORFs by analyzing the non-synonymous to synonymous substitution ratios (*d*_N_/*d*_S_) (Fig. 5b). Purifying selection was detected at branches *a* and *c* of the phylogenetic tree in Fig. 5b (branch *a*: *d*_N_/*d*_S_ ratio = 0.40, likelihood ratio test (LRT): *p* < 0.05; branch *c*: *d*_N_/*d*_S_ ratio = 0.25, LRT: *p* < 0.01). Taken together, these results indicate that natural selection occurred during eEBLL-1 evolution, suggesting that eEBLL-1 was exapted as a functional protein in the bat species.

### Phylogenetic analysis

**Figure 6:**
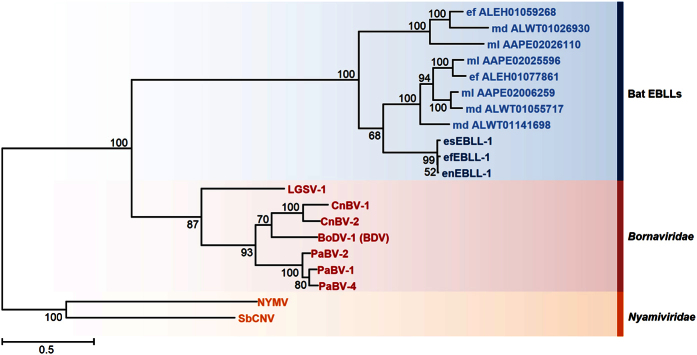
A phylogenetic tree of bat EBLLs with bornaviral and nyamiviral L genes. The JTT + G model of amino acid substitution was used in the construction of the maximum likelihood tree. The bootstrap value is indicated for each interior branch. The scale bar shows the number of amino acid substitutions per site. eEBLL-1s are indicated in dark blue. Other EBLL elements in bat genomes and their accession numbers are shown in light blue. ml, *M. lucifugus*; md, *M. daubentonii*, ef, *E. fuscus*, LGSV-1, Loveridge’s garter snake virus 1; CnBV-1 and CnBV-2, canary bornaviruses; BoDV-1 (BDV), Borna disease virus; PaBV-1, -2, and -4, parrot bornaviruses; NYMV, Nyamanini nyavirus; SbCNV, Soybean cyst nematode virus.

To gain insights into the evolution of mononegaviral polymerases, we constructed a phylogenetic tree from the amino acid sequences of bat EBLLs and the representative L genes of the families *Bornaviridae* and *Nyamiviridae*. The tree revealed three major clusters: bat EBLLs, exogenous bornaviral L genes, and nyamiviral L genes (Fig. 6). Together with Fig. S1, our data suggest that the bat EBLLs might be derived from ancient bornaviruses distantly related to known modern bornaviruses.

## Discussion

In this study, we discovered an endogenous RNA virus element that encodes a predicted RdRp gene of an ancient negative-strand RNA virus in bats of the genus *Eptesicus*. Notably, this element (eEBLL-1) contains a 1,718-amino acid ORF that was conserved for more than 11.8 MY and included almost all of the sequence motifs essential for the enzymatic activity of a RNA virus RdRp. To the best of our knowledge, eEBLL-1 is the first example of an RNA virus-derived RdRp encoded by the mammalian genome. Together with the data of the transcription profile and the natural selection of eEBLL-1, our observations strongly suggested that eEBLL-1 was exapted by the *Eptesicus* genomes as a functional protein, rendering a survival advantage to the bats. However, at present its evolutionary significance for the host, if any, is unclear. Based on the knowledge of EVE-derived genes, we propose two hypotheses. One possible function of the eEBLL-1s is to affect the replication of exogenous bornavirus-related viruses. EVE-derived immunity (EDI) is a well-known concept because EVEs act as anti-viral genes against genetically similar viruses^27^. Indeed, we have demonstrated that the expression of itEBLN inhibits BDV replication^13^ and that some EBLNs in rodent and primate genomes express piRNAs that may interfere with bornavirus infection^16^. The successful infection and replication of bornaviruses could be accomplished by the balanced activity of RdRp in infected cells. Thus, eEBLL-1 might protect its hosts from related viruses by perturbing their replication balance via its RdRp activity. Alternatively, enhanced viral replication may stimulate host immune responses. Indeed, BDV antigen expression was reported to be enhanced by exogenous expression of the BDV L protein^26^. Therefore, excess RdRp activity might cause the dysregulation of transcription/replication, resulting in the disruption of infection. To investigate whether efEBLL-1 affects BDV polymerase activity, we conducted a minireplicon assay for BDV using recombinant eEBLL-1. The result showed that the expression of esEBLL-1 did not have an effect on the polymerase activity of BDV (Supp. Fig. 8). However, we cannot exclude the possibility that eEBLL-1 affects the replication of bornaviruses with L genes that are genetically similar to eEBLL-1, although bornavirus has not yet been detected from bats. The phylogenetic analyses showed that BDV L is relatively distant from the eEBLL-1s (Fig. 6 and Supp. Fig. 1). Bornavirus species may exist that are genetically much closer to eEBLL-1 in the bat species. Another hypothesis is that eEBLL-1 might be involved in the RNA interference (RNAi) machinery of the host cells as an RdRp. At present, several eukaryotic proteins have been reported to act as RdRps^28^,^29^,^30^. Among these proteins, the RNA-directed RNA polymerases (RDRs) are well-studied, authentic RdRps that are encoded by several eukaryote species, such as plants, nematodes and fission yeasts^30^. RDRs are essential for the RNAi machinery, which is involved in anti-viral defence and gene regulation in these species. Interestingly, although RDR genes are derived from common ancestral genes, higher eukaryotes have lost RDR genes during evolution^30^, although they still retain the RNAi machinery for anti-viral defense^31^,^32^. Although the mononegaviral L proteins require the N and P proteins for their polymerase activities, the L protein of vesicular stomatitis virus has been reported to replicate RNA from an RNA template in the absence of the viral proteins N and P^33^. Thus, the eEBLL-1s may function as RdRps in the absence of other viral proteins. The eEBLL-1-mediated RNAi machinery could restrict viral replication of not only bornaviruses but also other viruses as previously reported in other viruses^31^,^32^ that may have conferred survival advantages to their host species. Further studies are needed to assess our hypotheses. Our findings provide novel insights into the co-evolution of RNA viruses and mammalian species.

## Methods

### Tissue samples

In Japan, the capture and handling of a bat were approved by the Japanese Ministry of Environment (license No. 21-27-0213 to 21-27-0215), and conducted in accordance with the approved protocol. An *E. nilssonii* was captured in Hokkaido, Japan and euthanized; then, the liver was removed. The liver sample was stored in RNAlater (Thermo Fisher Scientific, Waltham, MA, USA) prior to the isolation of genomic DNA. Bat muscle tissue (*E. serotinus*) and cell cultures from *E. serotinus*, *M. daubentonii* (MyDauNi/2), *M. nattereri* (MyLu/2), *Nyctalus noctula* (NyNoNi/2) and *Pipistrellus pipistrellus* (PipNi/1) were available from previous studies and originated from animals found dead in Germany and delivered to centres for bat protection^34^,^35^,^36^,^37^.

Because all German bats are protected through the European Commission (http://ec.europa.eu/environment/nature/legislation/habitatsdirective) and the Agreement on the Conservation of Populations of European Bats (www.eurobats.org), investigative research requires special permission by local government bodies. As part of a study on diseases in European bats^38^, 486 bats of nineteen vespertilionid bat species were examined. Most animals were found dead, injured or moribund near roosting sites or human habitations in urban and suburban areas of different regions in Germany. If the bats died in care or had to be euthanized for medical reasons, the carcasses were immediately stored at −20 °C and transferred to the Leibniz Institute for Zoo and Wildlife Research (Berlin, Germany) for pathological, histopathological and bacteriological examinations. Subsequently, aliquots of the individual organs were sent to the Robert Koch Institute for virological examination.

### PCR screening of EBLL elements

DNA samples were extracted using the QIAamp DNA Blood Mini Kit (QIAGEN, Hilden, Germany), Quick-gDNA MiniPrep kit (Zymo Research, Irvine, CA, USA) or NucleoSpin Tissue kit (Macherey & Nagel, Düren, Germany). To detect EBLL elements, a PCR was performed in a final volume of 50 μl containing 1.25 U of Ex Taq polymerase (TaKaRa), 1x Ex Taq buffer, 0.2 mM dNTPs, 0.5 M Betaine, 0.4 μM primers and 10 ng of gDNA as a template. The primer sets MH185–186, 187–188 and 189–190 were used for the detection of EBLL sequences (primer sequences are available in Supp. Table 3). The PCR conditions were as follows: denaturation at 94 °C for 2 min, 40 cycles of 94 °C for 30 sec, 60 °C for 30 sec, and 72 °C for 30 sec, followed by a final extension at 72 °C for 3 min. Amplified DNA was analysed by agarose gel electrophoresis.

### Detection of eEBLL-1 transcripts

To detect eEBLL-1 transcripts, RNA samples were isolated from bat brain tissues preserved in RNAlater (*Eptesicus nilssonii* [n = 5] and *Eptesicus serotinus* [n = 5]). The RNA extraction was performed using the NucleoSpin RNA Extraction Kit (Macherey & Nagel, Düren, Germany). For cDNA synthesis, we utilized the TaqMan Reverse Transcription Reagents kit (Thermo Fisher Scientific) with an additional denaturing step of 95 °C for 5 min using oligo-dT primers. Bat species were determined by amplification and sequencing of mitochondrial DNA as described by Sonntag *et al.*^39^. PCR was performed using the Premix Ex Taq Hot Start Version (TaKaRa) in a final volume of 50 μl containing 0.4 μM primers and 1 μl of cDNA. The primer sets MH185-186, 187–188 and 189–190 were used for the detection of EBLL sequences (primer sequences are available in Supp. Table 3). The PCR conditions were as follows: denaturation at 94 °C for 2 min, 35 cycles of 94 °C for 30 sec, 60 °C for 30 sec, and 72 °C for 30 sec, followed by a final extension at 72 °C for 3 min. The PCR products were analysed by electrophoresis.

### BLAST search

A tBLASTn search was conducted using the nucleotide sequence of efEBLL-1 (ALEK01070252) as a query on 4 June 2013. The whole genome shotgun sequences (WGS) of *E. fuscus*, *M. davidii*, and *M. lucifugus* were selected as the databases. Sequence hits with e-values <10^−20^ and their surrounding regions alignable to efEBLL-1 were considered homologous to efEBLL-1. The transposable elements (TEs) in bat WGS contigs were identified by CENSOR software^40^.

### Amino acid sequence analyses

Amino acid sequences of bornaviral Ls and eEBLL-1s were aligned using the PSI-Coffee program^41^. Functional domain searches were conducted by Pfam^42^. The conservation of functional motifs among mononegaviral RdRps was determined by a manual search.

### Phylogenetic analyses

Most of the bat EBLLs identified by the tBLASTn search were fragmentary, probably due to the occurrence of point mutations, insertions/deletions, and genome rearrangements after the integration of the viral L genes (Supp. Fig. 3). Neighbouring fragments were considered to be derived from a single integration event and combined into a single EBLL sequence if the number of overlapping amino acid positions between the regions in the efEBLL-1 sequence that were identified to be homologous to the fragments was smaller than 10. Parts of fragments homologous to the overlapping positions were not included in the combined sequence. Multiple alignments of (combined) amino acid sequences for bat EBLLs were constructed by mapping each EBLL sequence onto the efEBLL-1 sequence according to the pairwise alignment of the sequences produced in the tBLASTn search described above. Additionally, a multiple alignment of viral L protein sequences was generated by MAFFT^43^. Finally, multiple alignments of bat EBLLs and viral Ls were joined according to the pairwise alignment of efEBLL-1 (included in the bat EBLLs) and the BDV L of strain He/80/FR (included in the viral L gene) that were produced in the tBLASTn search, which identified efEBLL-1 as a homologue of the query He/80/FR L^12^; gaps were introduced at the same sites in all sequences in either alignment to maintain the relationship when joining the alignments. Phylogenetic trees for the amino acid sequences of the bat EBLLs and viral Ls were constructed by the maximum likelihood method with the pairwise deletion option in MEGA6^44^. The JTT + G and LG + G models were judged to be the fittest for the data because they had the lowest Bayesian information criterion scores. The reliability of each internal branch in the phylogenetic tree was assessed by computing the bootstrap probability with 100 resamplings.

### Natural selection operating on eEBLL-1

The *d*_N_/*d*_S_ ratio at each branch of the phylogenetic tree for the eEBLL-1s was estimated by the maximum likelihood method using the codon substitution model in PAML ver. 4.0^45^. The topology was derived from the phylogenetic tree of bat EBLLs in Fig. 5b. The equilibrium codon frequencies were treated as free parameters, and the *d*_N_/*d*_S_ ratio was estimated under the free-ratio (selection) model and the branch-specific (null) model. The *d*_N_/*d*_S_ ratio was allowed to vary among branches in the selection model, whereas in the null model the *d*_N_/*d*_S_ ratio was fixed at 1 at specified branches. The null hypothesis of no selection (*d*_N_/*d*_S_ = 1) at the specified branches was tested by the likelihood ratio test.

### Simulation

The probability that efEBLL-1 and esEBLL-1 maintained the ORF under selective neutrality after the divergence of *E. fuscus* and *E. serotinus* was determined by simulating the evolution of efEBLL-1 and esEBLL-1 using Seq-Gen^46^. The nucleotide sequences of efEBLL-1 and esEBLL-1 were evolved according to the HKY model after removing termination codons with an evolutionary rate of 2.03 × 10^−9^ per site per year, which was estimated from the evolutionary distance at the 3rd codon position of the RAG2 exon and the divergence time of *E. fuscus* and *E. serotinus* (11.8 MYA in TimeTree^21^). The transition/transversion rate ratio was assumed to be 1.92, which was estimated from the 3rd codon position in the RAG2 exon of *E. fuscus*, *E. serotinus*, and *E. nilssonii* with the Tamura-3 parameter model by the maximum likelihood method. The distribution of the number of premature termination codons in the simulated sequences was obtained from 100,000 iterations.

### Minireplicon assay

We developed a pol II-driven firefly luciferase-based minireplicon assay for BDV (the construct is available upon request). For transfection, 2 × 10^5^ 293T cells were seeded into each well of a 12-well plate. The next day, plasmids expressing the BDV N, P and L proteins, the minigenome and Renilla luciferase (as a normalization control) with or without a plasmid expressing esEBLL-1 were transfected using Lipofectamine 2000 (Life Technologies). After 24 hours, the cells were lysed with Passive Lysis Buffer (Promega), and the luciferase activities were measured using the Dual-Luciferase Reporter Assay System (Promega). The values of firefly luciferase were normalized by the Renilla luciferase activity.

## Additional Information

**How to cite this article**: Horie, M. *et al.* An RNA-dependent RNA polymerase gene in bat genomes derived from an ancient negative-strand RNA virus. *Sci. Rep.* **6**, 25873; doi: 10.1038/srep25873 (2016).

## Supplementary Material

Supplementary Figures

Supplementary Tables
