# Supplementary material for: An RNA-dependent RNA polymerase gene in bat genomes derived from an ancient negative-strand RNA virus
Source: Sci Rep. 2016 May 13;6:25873. doi: 10.1038/srep25873 (PMC4865735; doi:10.1038/srep25873)
Supplement: Supplementary Figures [file srep25873-s1.pdf]

An RNA-dependent RNA polymerase gene in bat genomes derived from an ancient negative-strand RNA virus

Masayuki Horie*, Yuki Kobayashi, Tomoyuki Honda, Kan Fujino, Takumi Akasaka, Claudia Kohl, Gudrun Wibbelt , Kristin Mühldorfer, Andreas Kurth, Marcel A. Müller, Victor M. Corman, Nadine Gillich, Yoshiyuki Suzuki, Martin Schwemmle and Keizo Tomonaga*

*Corresponding authors:
Dr. Masayuki Horie: Transboundary Animal Diseases Research Center, Joint Faculty of Veterinary Medicine, Kagoshima. University, Kagoshima 890-0065, Japan. Email: mhorie@vet.kagoshima-u.ac.jp
Dr. Keizo Tomonaga: Department of Viral Oncology, Institute for Virus Research, Kyoto University, Kyoto 606-8507, Japan. Email: tomonaga@virus.kyoto-u.ac.jp

Supplementary Figures 1- 9.

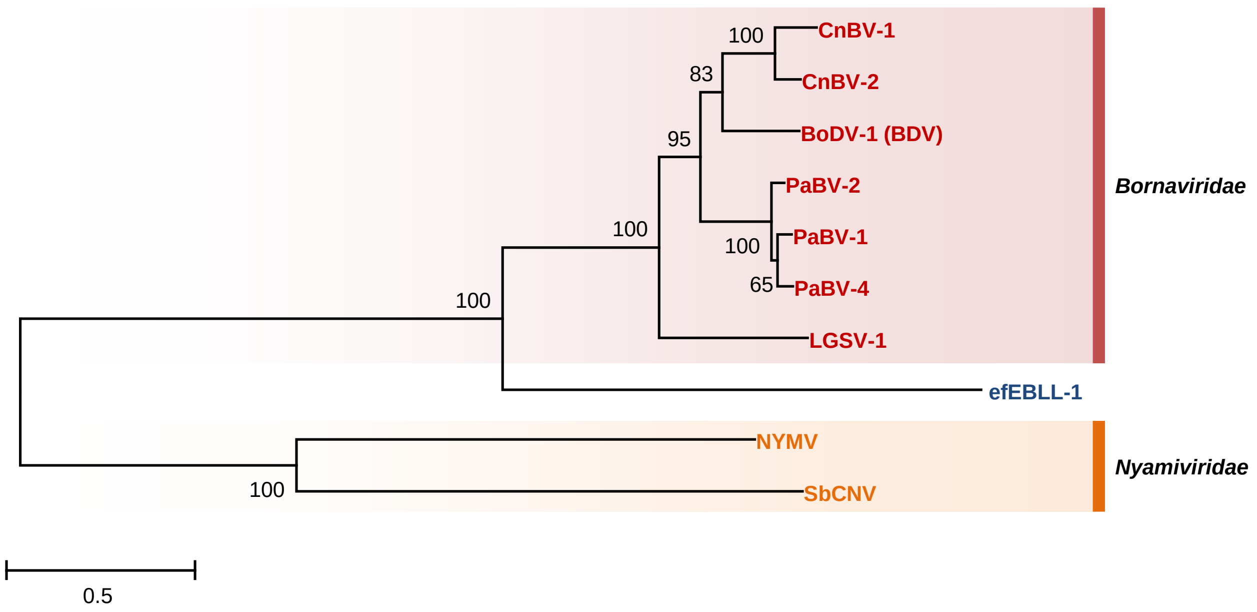

Supplementary Figure 1. A phylogenetic tree of efEBLL-1 and related viral L genes. The multiple alignment was made for amino acid sequences of bornaviral and nyamiviral L genes and efEBLL-1. LG+G model of amino acid substitution was used in the construction of the maximum likelihood tree. The reliability of the interior branch in the phylogenetic tree was assessed by the bootstrap method with 100 resamplings. CnBV-1 and -2, canary bornavirus; BoDV-1 (BDV), Borna disease virus; PaBV-1, -2 and -4, parrot bornavirus; LGSV-1, Loveridge's garter snake virus 1; SbCNV, Soybean cyst nematode virus; NYMV, Nyamanini nyavirus.

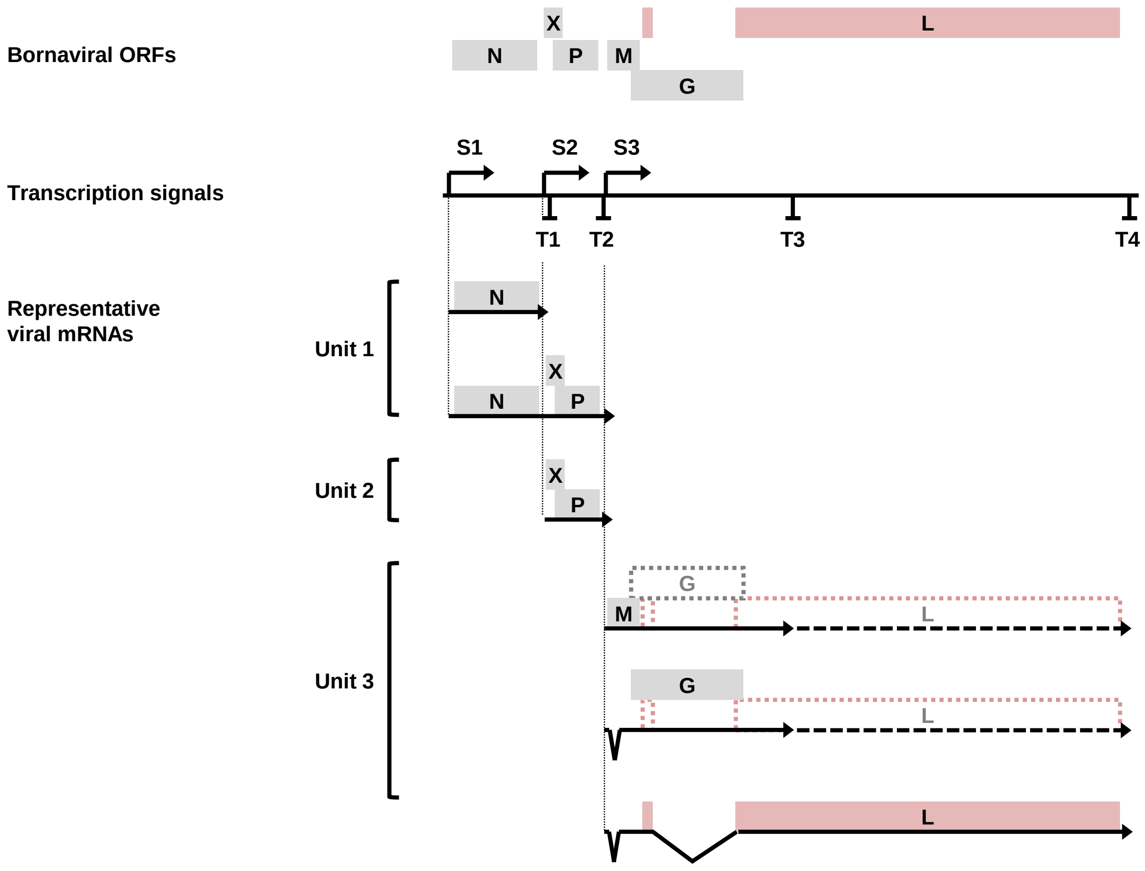

Supplementary Figure 2. Genome organization and transcripts of bornaviruses. Schematic figure of genome organization and transcripts of bornaviruses. The ORFs, transcription start signals (S1 – S3), transcription termination signals (T1- T4) and representative mRNAs of bornaviruses are shown. L gene is highlighted with red. Dashed arrows indicate read-through transcripts encoding M or G protein.

Supplementary Figure 3

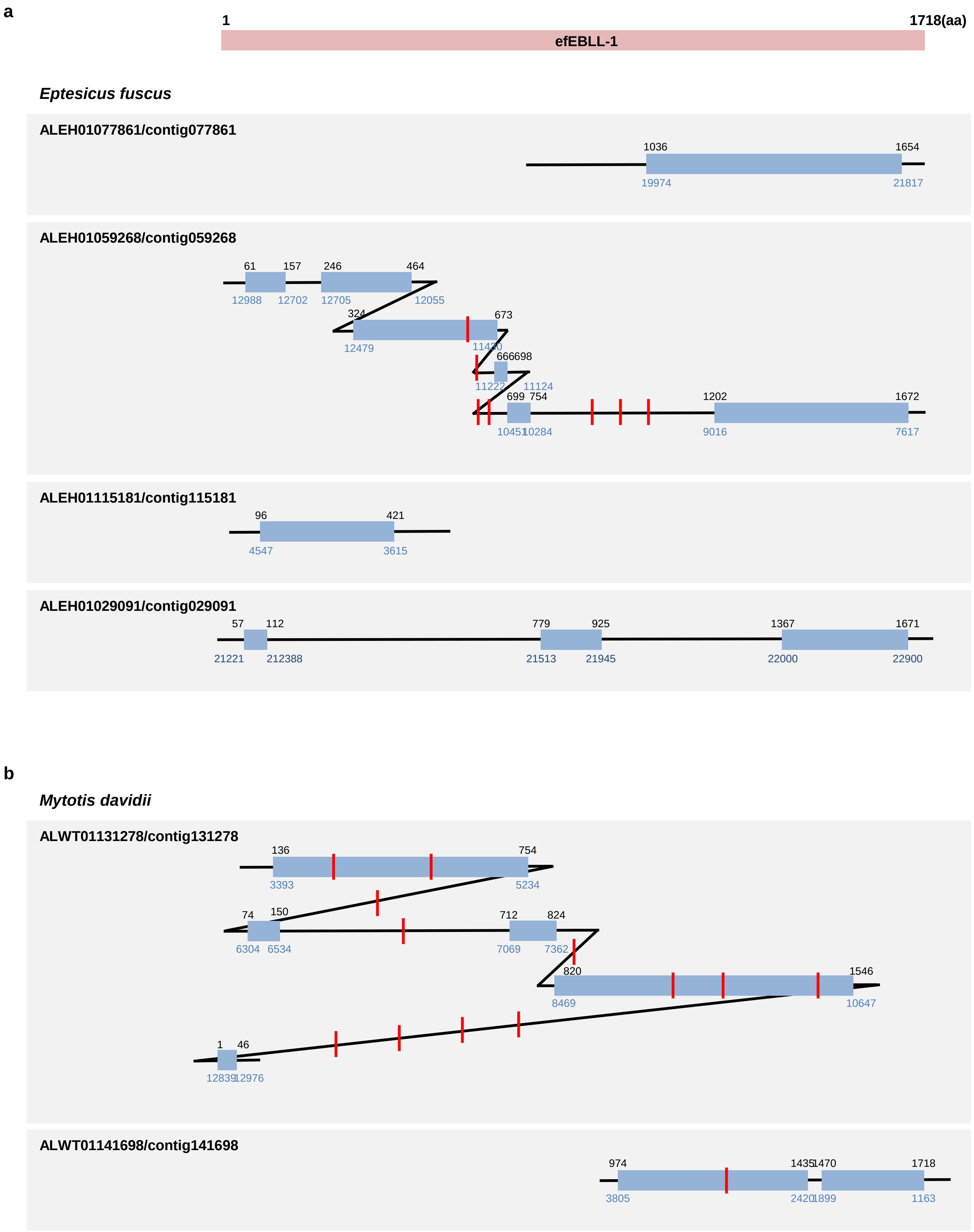

Supplementary Figure 3 (continued)

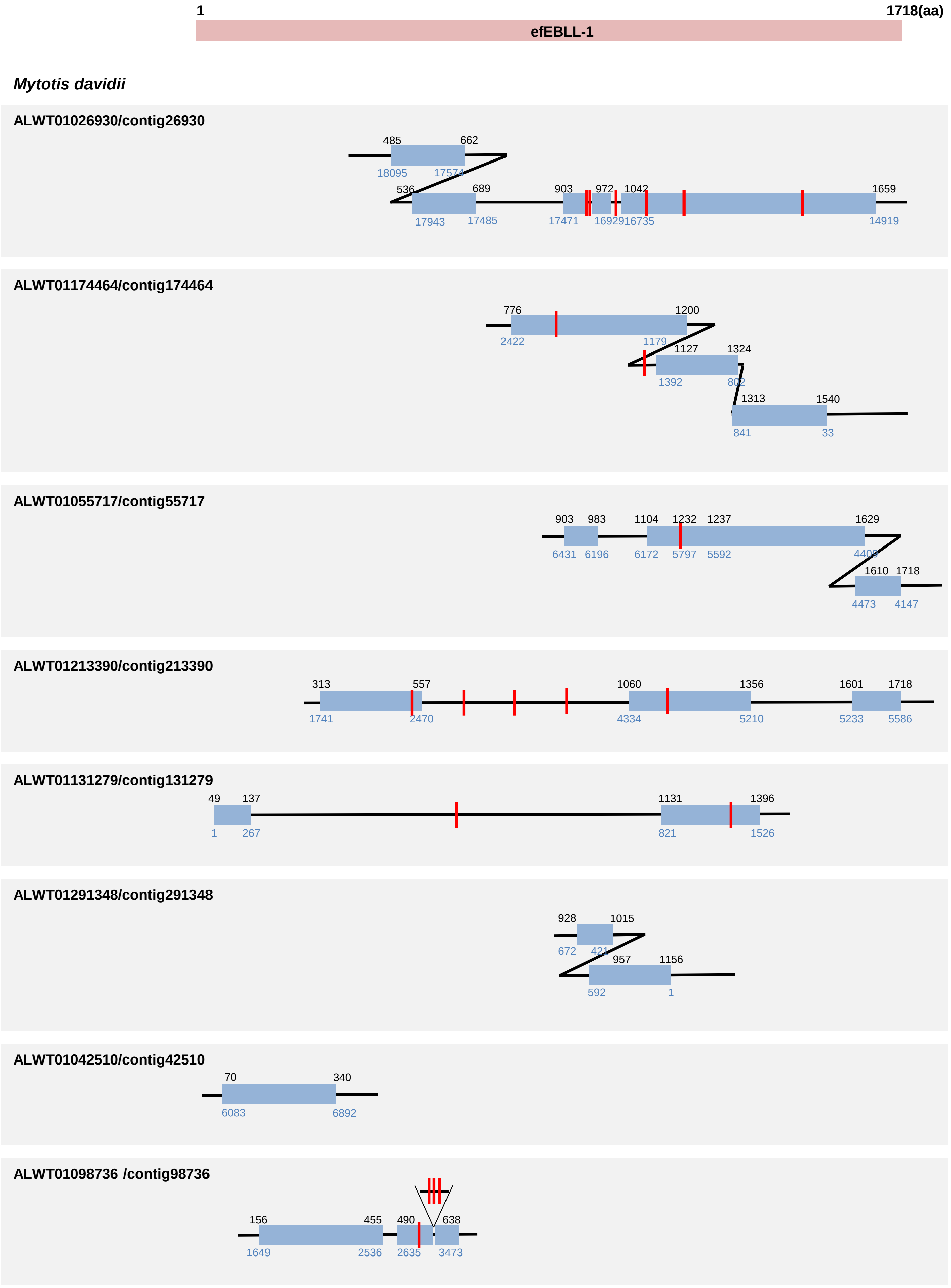

Supplementary Figure 3 (continued)

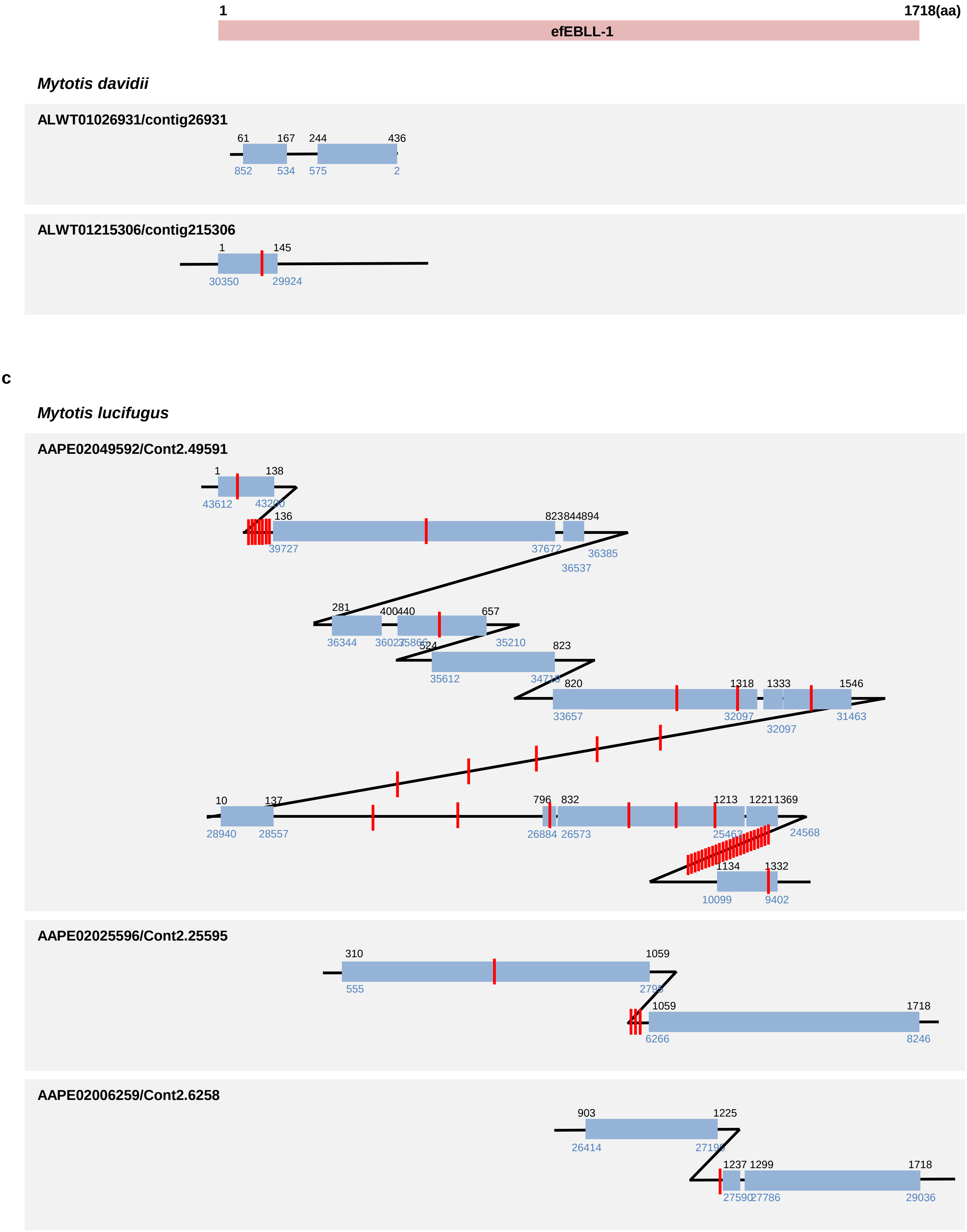

Supplementary Figure 3 (continued)

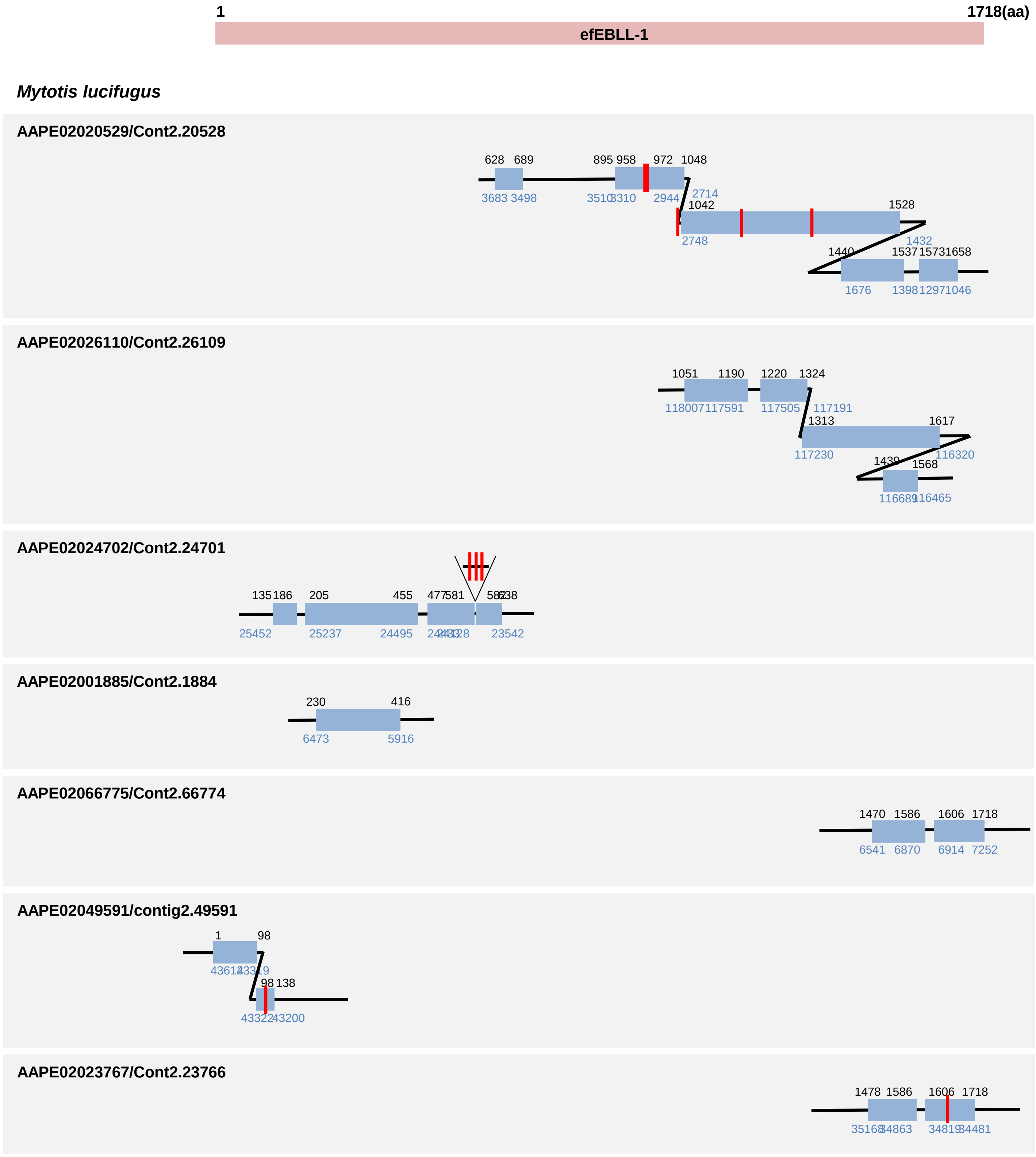

Supplementary Figure 3. The structures of EBLL elements in bats belonging to the family *Vespertilionidae*. Schematic diagrams of the structure of EBLL elements in *Eptesicus fuscus* (**a**), *Mytotis davidii* (**b**) and *M. lucifugus* (**c**). The EBLL elements were searched by tBLASTn using the deduced amino acid sequence of efEBLL-1 as a query. Black and blue numbers surrounding blue boxes indicate the amino acid positions in efEBLL-1 and the nucleotide positions in the bat whole genome shotgun contigs, respectively. Blue boxes show regions homologous to efEBLL-1. Red lines represent transposable elements.

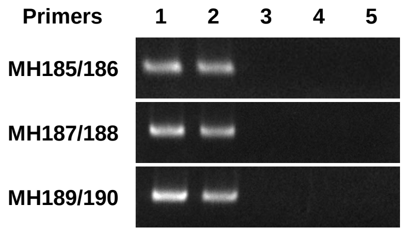

Supplementary Figure 4. Detection of EBLL elements in bat genomes. PCR amplification of EBLL elements from genomic DNAs with primer pairs MH185-186, MH187-188 or MH189-190. 1. *Eptesicus serotinus*; 2. *E. nilssonii*; 3. *Pipisterillus spec.*; 4. *Nyctalus noctula*; 5. *Myotis daubentonii*.

a

```
                                           TSD
efEBLL-1  -367:GCCATTTGGTTGATTTGCATATTAATCTTTTTTTTTTCCCCCATTCTTGCTGTAAATTTGCCACAAGGTA -298
enEBLL-1  -367:GCTGTTTGGTTGATTTGCATATTAATCTTTTTTTTTCCCCCCATTCTTGCTGTAAGCTTGCCACAAGGTA -298
esEBLL-1  -365:GCCGTTTGGTTGATTTGCATATTAATCTTTTTTTTT--CCCCGTTCTTGCTGTAAGCTTGCCACAAGGTA -298
               **  *******************************   **** ************  *************

          -297:TTGAGAATTGCGCTGGCAAGCTCAAAGACGTTCATTAGATTGAGATCTGCAATTCAGCTCATCATGATTG -228
          -297:TTGAGAATTGCGCTGGCAAGCTCAAAGACGTTCATTAGATTGAGATCTGCAATTCAGCTCATCATGATTG -228
          -297:TTGAGAATTGCGCTGGCAAGCTCAAAGACGTTCATTAGATTGAGATCTGCAATTCAGCTCATCATGATTG -228
               **********************************************************************

          -227:ATTTTGAACCAATCAGTGCACTCAATGCAGGTCTCTTTTACAGGTGGAGTGACCAAGCTTACCGTTGCCT -158
          -227:ATTTTGAACCAATCAGTGCACTCAATGCAGGTCTCTTTTACAGGTGGAGTGACCAAGCTTACCGTTGCCT -158
          -227:ATTTTGAACCAATCAGTGCACTCAATGCAGGTCTCTTTTACAGGTGGAGTGACCAAGCTTACCGTTGCCT -158
               **********************************************************************

          -157:GAAACTGAGTCTTGTTTCACCTGACAAGTTTCCCAAGCAAGGGAGAATTGATCTATCAACTGAAATAAGT -88
          -157:GAAACTGAGTCTTGTTTCACCTGACAAGTTTCCCAAGCAAGGGAGAATTGATCTATCAACTGAAATAAGT -88
          -157:GAAACAGAGTCTTGTTTCACCTGATAAGTTTCCCAAGCAAAGGAGAATTGATCTATCAACTGAAATAAGT -88
               ***** ****************** *************** *****************************

           -87:CGTGATTTGCTATCGTCCCAATTTGAGGTGGAGTCCTACAAGCTTGAATTATAGGGCCAAGACCTCTTGG -18
           -87:CGTGATTTGCTATCGTCCCAATTTGAGGTGGAGTCCTACAAGCTTGAATTATAGGGCCAAGACCTCTTGG -18
           -87:CGTGATTTGCTATTGTCCCAATTTGAGGTGGAGTCCTACAAGCTTGAATTATAGGGCCAAGACCTCTTGG -18
               ************* ********************************************************
                                 Start codon
           -17:AATTTATATGCGACGTCATGGCAATGTACCGTATGAACCAAGTCCTGAAGTCACCGCTTCTGGGAACTGA 53
           -17:GATTTATATGCGACATCATGGCAATGCACCGTATGGACCAAGTCCTGAAGTCACCGCTTCTGGGAACTGA 53
           -17:GATTTATATGCGACATCATGGCAATGCACCGTCTGGACCAAGTCCTGAAGTCACCGCTTCTGGGAACTGA 53
                ************* *********** ***** ** **********************************
```

b

```
                                         Stop codon                  T4 / poly-A
efEBLL-1  5128:TCTCTACATCATATTGCACACTTGCTCTAGGTAGCCCTACCGTGACATGGTAATATGATTAAAGAAAAAC 5197
enEBLL-1  5128:TCTCTACATCATATTGCACACTTGCTCTAGGTAGCCCTACCATGACGTGGTAATAGGATTAAAGAAAAAC 5197
esEBLL-1  5128:TCTCTACATCATATTGCACACTTGCTCTAGGTAGCCCTACTGTGACGTGGTAATATGATTAAAGAAAAAC 5197
               ****************************************  **** ******** **************
                 TSD
          5198:ATATTAATAGGTCATTAACCCCCCTTTTTAAATTTGTTTATTGAGTATAGATCCATGTCTTTATGACTTA 5267
          5198:ATATGAATAGGTCATTAACCCCCCTTTTTTTATTTGTTTATTGAGTATAGATCCATGTCTTTATGACTTA 5267
          5198:ATATTAATAGGTCATTAACCCCCCTTTTTTTATTTGTTTATTGAGTATAGATCCATGTCTTTATGACTTA 5267
               **** ************************  ***************************************

          5268:TGTCTCATGTCTCATTCATTAGGTGCACTAAAATGCCCCCCAGCCTGAGATCCACTTGCTATACTGACAG 5337
          5268:TGTCTCATGTCTCATTCATTAGGTGCACTAAAATGCCCCCCAGCCCGAGATCCACTTGCTATACTGACAG 5337
          5268:TGTCTCATGTCTCATTCATTAGGTGCACTAAAATGCCCCCCAGCCCGAGATCCACTTGCTATACTGACAG 5337
               ********************************************* ************************
```

Supplementary Figure 5. Alignment of efEBLL-1, enEBLL-1, esEBLL-1 and their flanking sequences. Nucleotide alignments of 5’ (**a**) or 3’ regions (**b**) of EBLL-1s. Blue boxes indicate the ORFs. Start and stop codons are underlined. Blue letters indicates transcription termination signal (T4)-like and poly-A sequences. TSD, target site duplication.

Supplementary Figure 6

a

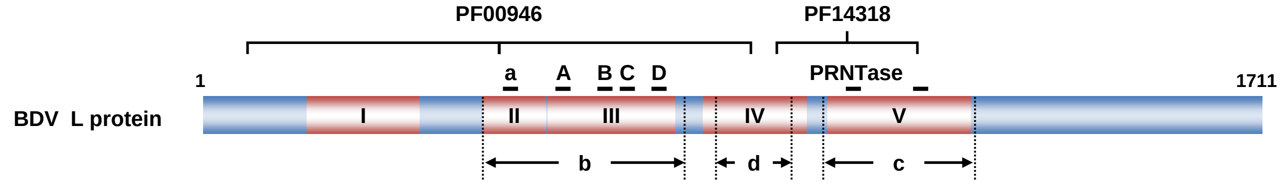

b

```
                                                               a
ABV        440:PSRLVNALIDGKLDSIPELLEPFYKGSVNFDDRITVLVPKEKELKVKGRFFSKQSLAIRIYQVVAEASIKNEIMPYLKTH 519
BDV        440:PSRLVNALIDGHLDNIPALLEPFYRGAVEFEDRLTVLVPKEKELKVKGRFFSKQTLAIRIYQVVAEAALKNEVMPYLKTH 519
efEBLL-1   430:VQRLILALLRGDLDDIKGQLQPFMTGMISRKDLITVLVPKEKELKVKGRYFPKQSCRIRLYQVLAELNLKKFIMPYLRMH 509
enEBLL-1   430:VQRLILALLRGDLDDIKGQLQPFMTGMISRKDLITVLVPKEKELKVKGRYFPKQSCRIRLYQVLAELNLKKFIMPYLRMH 509
esEBLL-1   430:VQRLILALLRGDLDDIKGQLQPFMTGMISRKDLITVLVPKEKELKVKGRYFPKQSCRIRLYQVLAELNLKKFIMPYLRMH 509
                 **  **  * ** *   * **  *    *  ******************* * **   ** *** **   *   ****  *
                                                A
           520:SMTMSSTSLTHLLNKLSNLIVNGDSFVINLDYSSWCNAFRPELQMPLCRQLDQMFDCGFFFRTGCILPCFTTFIIQDRFN 599
           520:SMTMSSTALTHLLNRLSHTITKGDSFVINLDYSSWCNGFRPELQAPLCRQLDQMFNCGYFFRTGCTLPCFTTFIIQDRFN 599
           510:SMTTSSTQLSHILDRISSLLSRGNRFIINLDYESWSSTFRPELQDPLCRELDRMFGSDCFYQVGSWMPSITTFIMQDRFN 589
           510:SMTTSSTQLSHILDRISSLLSRGNRFIINLDYESWSSTFRPELQDPLCRELDRMFGSDCFYQVGSWMPSITTFIMQDRFN 589
           510:SMTTSSTQLSHILDRISLLLSRGNRFIINLDYESWSSTFRPELQDPLCRELDRMFGSDCFYQVGSWMPSITTFIMQDRFN 589
               *** *** * * *   *     *  * ***** **   ****** **** ** **    *   *   *  **** *****
                                          B                              C
           600:PPAADIRTLPVEDMVTCAHGTKTMGEGMRQKLWTILTSCWEIMALREAQVSFNILGQGDNQTIIVYRSITETNQALADRA 679
           600:PPYSFR-GEPVEDGVTCAVGTKTMGEGMRQKLWTILTSCWEIIALREINVTFNILGQGDNQTIIVHKSASQNNQLLAERA 678
           590:PPRQDDNGYPCDDMETCAQGTTTMGEGMRQKLWTILTACLELVTLEAVGIHGEVLGQGDNQTILITPPRHMSQEDARDVV 669
           590:PPRQDDNGYPCDDMETCAQGTATMGEGMRQKLWTILTACLELVTLEAVGIHGEVLGQGDNQTILITPPRHMSQEDARDVV 669
           590:PPRQDDNGYPCDDMETCAQGTATMGEGMRQKLWTILTACLELATLEAVGIHGEVLGQGDNQTILITPPRHMSQEDARDVV 669
               **       *  *  *** ** *************** * *   *         *********

           680:LGCLYKYARLAGHTLKVEECWVSDCLYEYGKRLFFEGIPIPGSLKQLSRVTDSTGELFPNLYSKLACLVSS          750
           679:LGALYKHARLAGHNLKVEECWVSDCLYEYGKKLFFRGVPVPGCLKQLSRVTDSTGELFPNLYSKLACLTSS          749
           670:IKHLQQRSHEAGLVLKPEECWSSDILYEYGKKLYYDGVPVPNFLKIFSRISDSTGEVYPNVYSRLACLSSS          740
           670:IKHLQQRSHEAGLVLKPEECWSSDILYEYGKKLYYDGVPVPNFLKIFSRISDSTGEVYPNVYSRLACLSSS          740
           670:IKHLQQRSHEAGLVLKPEECWSSDILYEYGKKLYYDGVPVPNFLKIFSRISDSTGEVYPNVHSRLACLSSS          740
                  *      **  ** **** ** ****** * *   * * *  **  **  *****  **  * **** **
```

c

```
                                                                            PRNTase
ABV        991:LIGCTYVAAKRLRRLTWGRDLIGVTMPFVAEQFNPVNSSNASLENYKDAILYVPQEPLRERHLYTRGNQPLYLGSNTAIK 1070
BDV        990:LEGCTYLAAKQLRRLTWGRDLVGVTMPFVAEQFHPHSSVGAKAELYLDAIIYCPQETLRSHHLTTRGDQPLYLGSNTAVK 1069
efEBLL-1   982:LQACSVVAADKLRQDTWGVKLEGVSMPFIAEQFHLQET--ATSEEVACSIIFRPDTKLFPRQWTKRGSRPLYIGSRTHIK 1059
enEBLL-1   982:LQGCSVVAADKLRQDTWGVKLEGVSMPFIAEQFHLQET--ATSEEVARSIIFRPDTKLFPRQWTKRGSRPLYIGSRTHIK 1059
esEBLL-1   982:LQGCSVVAADKLRQDTWGVKLEGVSMPFIAEQFHLQET--ATSEEVARSIIFRPDTKLFPRQWTKRGSRPLYIGSRTHIK 1059
               *  *   **  **  ***  * ** *** ****       *  *     *   *   *       **  *** ** *  *
                                                                      PRNTase
          1071:VQRGELTGLSKSRAAGLVRDTLILYQWYKVRKVIDPNLNKLMDRFLQEKGYVSDARPVVHGGTLTHRLPSRGDSRQGLTG 1150
          1070:VQRGEITGLTKSRAANLVKDTLVLHQWYKVRKVTDPHLNTLMARFLLEKGYTSDARPSIQGGTLTHRLPSRGDSRQGLTG 1149
          1060:VNRGAITGLPEGKIGKMVEDLILTCNWIKLRGSVGGNMANLLLQLLKEKGVTSPTLPVIMGGTLTHRLPTASDDRAGLAG 1139
          1060:VNRGAITGLPEGKIGKMAEDLILTCNWIKLRGSVGGNMANLLLQLLKEKGVTSPTLPVIMGGTLTHRLPTASDDRAGLAG 1139
          1060:VNRGAITGLPEGKIGKMAEDLILTCNWIKLRGSVGGNMANLLLQLLKEKGVTSPTLPVIMGGTLTHRLPTASDDRAGLSG 1139
               * **  ***          *      * * *         *    * ***  *   *   *********   * * ** *

          1151:YVNLISTWLKFSSDYMSTYSQSSDDYTIHFQHVLTYGCLYADVVVRSGRVVNEPYLLTASCHECFEKIES          1220
          1150:YVNILSTWLRFSSDYLHSFSKSSDDYTIHFQHVFTYGCLYADSVIRSGGVISTPYLLSASCKTCFEKIDS          1219
          1140:NLNVISTHINFTTNYMTNFTKSQEDYTIHFQAAFLHGLNLLASKLHYGTLEPITYYLTTSCKTCTSLIEE          1209
          1140:NLNVISTHINFTTNYMTNFTKSQEDYTIHFQAAFLHGLNLLASKLHYGTLEPITYYLTTSCKTCTSLIEE          1209
          1140:NLNVISTHINFTTNYMTNFTKSQEDYTIHFQAAFLHGLNLLASKLHYGTLEPITYYLTTSCKTCTSLIEE          1209
                 *  **   *   *      *  *******     *          *      * *  **  *   *
```

d

```
                                                      NLS
ABV        809:TLPSVFFRGMSDPLPLQLALLKTLVRLTGVSIEFVNSVVKLRIAAYPDWLSLITDPTSLNIQQLLRPERQIRRWVEQAIL 888
BDV        808:TLPSVFFRGMSDPLPFQLALLQTLIKTTGVTCSLVNRVVKLRIAPYPDWLSLVTDPTSLNIAQVYRPERQIRRWIEEAIA 887
efEBLL-1   799:TLPSVMLRGVPDPLTFQLSLLKSAISI-GVSKSCIHQVAKLQIPNEPSALALCSDPTCLNIIPLRRPEIVLKTWIEESLS 877
enEBLL-1   799:TLPSAMLRGVPDPLTFQLSLLKSAISI-GVSKSCIHQVAKLQIPNEPRALALCSDPTCLNIIPLRRPEIVLKTWIEESLS 877
esEBLL-1   799:TLPSVMLRGVPDPLTFQLSLLKSAISV-GVSKSCIHQVAKLQIPNEPRALALCSDPTCLNIIPLRRPEIVLKTWIEESLS 877
               ****   **  ***  ** **       **       * ** *   *  * *  *** ***    ***     * *
```

Supplementary Figure 6. Amino acid sequence alignments of bornaviral L and eEBLL-1s. a, Schematic figure of the BDV L protein. The numbers above the boxes indicate the amino acid residue positions. The pink boxes show the conserved blocks I-V of mononegaviral RdRp. The highly conserved stretches "a" and A-D, and the PRNTase domain are shown. The regions of the possible functional domains in eEBLL-1 by Pfam search (PF00946, Mononegavirales RNA dependent RNA polymerase domain and PF14318, Mononegavirales mRNA-capping region V domain) are shown. The regions shown in b, c and d are indicated. **b, c** and **d**, Amino acid sequence alignments spanning the box II and III (**b**), box V (**c**) and NLS region (**d**) of ABV L, BDV L and eEBLL-1s. Orange boxes indicate strictly invariant amino acid residues among the RNA-dependent polymerases. Red and green letters show functional motifs of mononegaviruses' RdRps (**b** and **c**) or NLS sequence of BDV (**d**), and highly conserved amino acid residues among the mononevaviral RdRps, respectively.

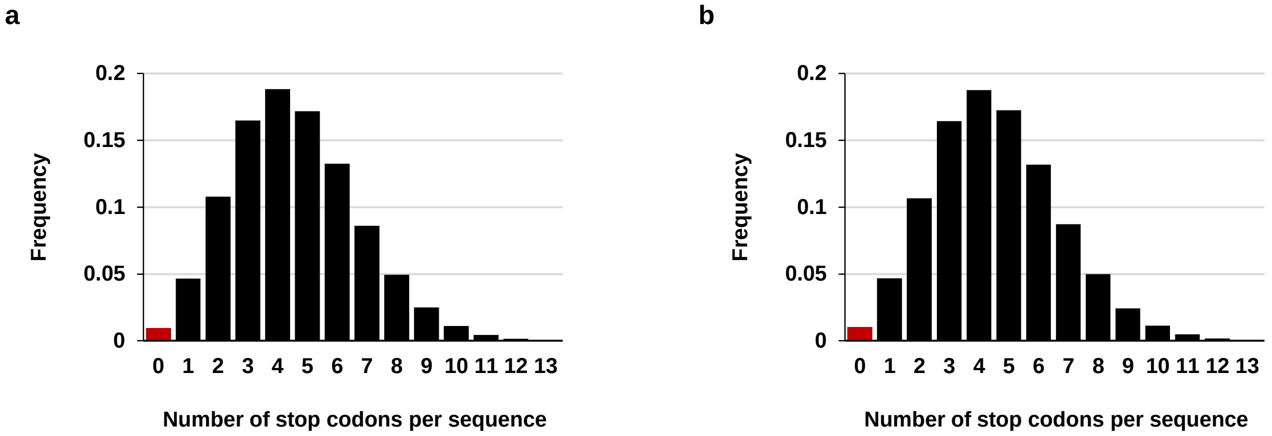

Supplementary Figure 7. Detection of natural selection in eEBLL-1. The distribution of the number of premature stop codons that enEBLL-1 (**a**) or esEBLL-1 (**b**) acquired during 11.8 million years under neutral evolution from 100,000 simulation replicates. The frequencies of zero stop codons are indicated by red bars [0.0097 (**a**) and 0.0103 (**b**)].

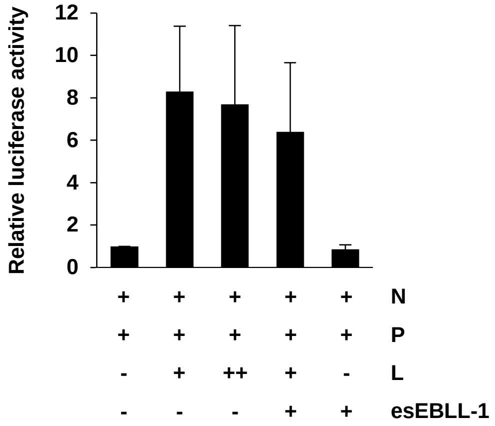

Supplementary Figure 8. eEBLL-1 does not affect polymerase activity of BDV. Minireplicon assays were performed in the presence or absence of esEBLL-1. Plasmids used for the assays were shown as “+” or “++”. “++” indicates double amount of the plasmid compared to “+”.

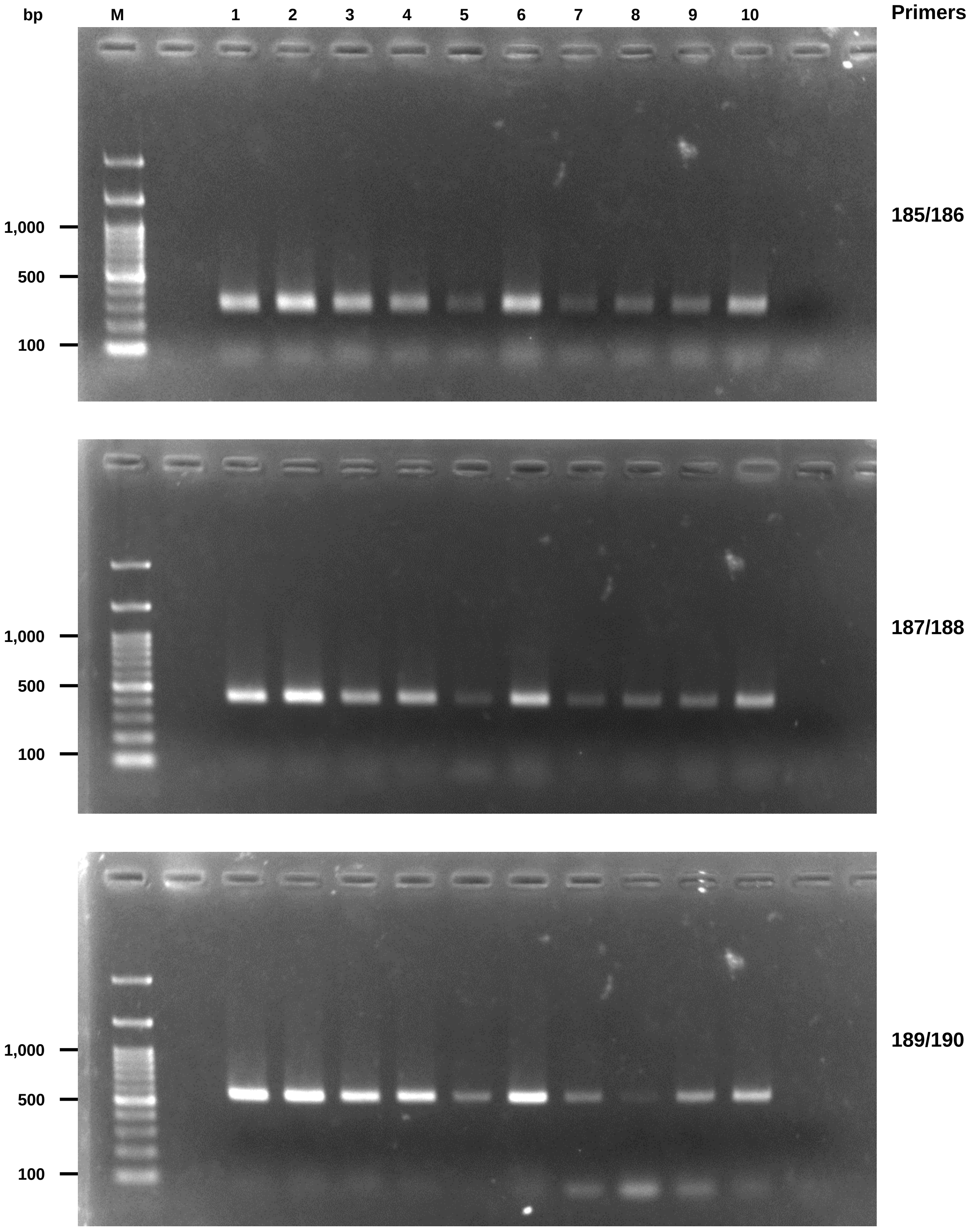

Supplementary Figure 9. Full-length gel images of Figure 4b. Primer pairs are indicated at the right. Lanes 1-5. *E. serotinus*; 6-10. *E. nilssonii*; 11. *M. daubentonii*. M, 100 bp radder marker.
