# Supplementary material for: An RNA-dependent RNA polymerase gene in bat genomes derived from an ancient negative-strand RNA virus
Source: Sci Rep. 2016 May 13;6:25873. doi: 10.1038/srep25873 (PMC4865735; doi:10.1038/srep25873)
Supplement: Supplementary Tables [file srep25873-s2.pdf]

An RNA-dependent RNA polymerase gene in bat genomes derived from an ancient negative-strand RNA virus

Masayuki Horie*, Yuki Kobayashi, Tomoyuki Honda, Kan Fujino, Takumi Akasaka, Claudia Kohl, Gudrun Wibbelt , Kristin Mühldorfer, Andreas Kurth, Marcel A. Müller, Victor M. Corman, Nadine Gillich, Yoshiyuki Suzuki, Martin Schwemmle and Keizo Tomonaga*

*Corresponding authors:
Dr. Masayuki Horie: Transboundary Animal Diseases Research Center, Joint Faculty of Veterinary Medicine, Kagoshima. University, Kagoshima 890-0065, Japan. Email: mhorie@vet.kagoshima-u.ac.jp
Dr. Keizo Tomonaga: Department of Viral Oncology, Institute for Virus Research, Kyoto University, Kyoto 606-8507, Japan. Email: tomonaga@virus.kyoto-u.ac.jp

Supplementary Tables 1- 4.

Supplementary table 1 Reverse BLAST analysis

Name (strain)	Accesssion number	Region		Identities/ similarities (%)	*E*-value
		efEBLL-1	hit		
Avian bornavirus L (bil)	ACG59353	1...1710	18...1683	37.1/ 54.9	0
Avian bornavirus L (6609)	ACS32310	1...1710	18...1683	36.9/ 54.8	0
Avian bornavirus L (NM_06)	AEW69865	1...1692	18...1671	36.4/ 54.9	0
Avian bornavirus C1 (#7298)	AGJ74872	5...1714	22...1700	37.3/ 55.1	0
Avian bornavirus L (NM_01)	AER30492	1...1692	18...1671	36.4/ 54.8	0
Borna disease virus L (huP2br)	BAF46124	5...1585	22...1581	38.1/ 56.3	0
Avian bornavirus L (16234)	AFN70793	1...1709	18...1682	36.6/ 54.8	0
Avian bornavirus L (AG5)	AEG41944	1...1692	18...1671	36.3/ 54.5	0
Avian bornavirus L (NM_20)	AEW69871	1...1692	18...1671	36.3/ 54.5	0
Borna disease virus L (V/FR)	P52639	5...1585	22...1581	38.2/ 56.3	0
Avian bornavirus L (M15)	AEW69859	1...1692	18...1671	36.2/ 54.4	0
Borna disease virus L (H1766)	CAC70652	5...1585	22...1581	38.1/ 56.2	0
Borna disease virus L (Bo/04w)	BAF46117	5...1585	22...1581	38.1/ 56.1	0
Borna disease virus L (CRP3B)	AAM68145	5...1585	22...1581	37.7/ 55.5	0
Borna disease virus L (CRNP5)	AAM68151	5...1585	22...1581	37.7/ 55.5	0
Borna disease virus L (CRP3A)	Q8JMN0	5...1585	22...1581	37.7/ 55.5	0
Avian bornavirus L (M14)	AER30498	1...1692	18...1671	36.2/ 54.3	0
Borna disease virus L (He/80/FR)	CAC70645	5...1585	22...1581	37.6/ 55.4	0
Borna disease virus L (No/98)	CAC70659	5...1626	22...1613	37.3/ 55.1	0
Avian bornavirus L (M25)	AEG78314	1...1709	36...1700	36.5/ 54.3	0
Avian bornavirus L (#15864)	AGJ74903	5...1680	22...1658	37.2/ 54.7	0
Avian bornavirus L (062-CG)	AGW46941	5...1706	22...1693	37.2/ 54.5	0
Avian bornavirus L (16667a)	AFN70809	5...1300	22...1313	42.2/ 60.2	0
Avian bornavirus L (CT15)	ADU05398	1...1259	18...1273	42.6/ 60.7	0
Borna disease virus L (V)	AAA20228	102...1585	13...1478	39.0/ 56.9	0
Borna disease virus L[a]	BAG55458	102...1585	13...1478	38.8/ 56.9	0
Borna disease virus L[a]	AAA20667	138...1585	1...1428	38.4/ 55.9	0
Avian bornavirus (1034_1322)	ACJ71393	1...865	18...876	43.2/ 61.5	0
Avian bornavirus (1367)	ACJ71386	1...821	18...831	44.1/ 62.4	0
Avian bornavirus L (VS-4707)	AHD24432	5...606	22...615	39.4/ 58.5	5.00E-122
Avian bornavirus L (M24)	AEE69063	1...558	18...568	39.7/ 59.3	2.00E-115
Midway virus L (RML47153)	ACQ94979	160...1225	196...1283	26.7/ 44.6	6.00E-84
Nyamanini virus L (tick 39)	ACQ94985	73...1225	108...1283	25.0/ 43.7	3.00E-83
Soybean cyst nematode midway virus L[a]	AEF56729	120...1223	148...1303	26.1/ 43.2	3.00E-80
Farmington virus L (CT 114)	AGN91191	270...1209	350...1336	24.6/ 41.8	3.00E-56

Supplementary table 1 Reverse BLAST analysis (continued)

Name (strain)	Accesssion number	Region		Identities/ similarities (%)	*E*-value
		efEBLL-1	hit		
Farmington virus L (CT 114)	AGN91191	270...1209	350...1336	24.6/ 41.8	3.00E-56
Borna disease virus L (RX98)	AAO62365	5...343	22...354	33.3/ 50.8	5.00E-41
Rabies virus L (F04)	ACN65050	455...1216	530...1334	25.1/ 43.6	5.00E-38
Shimoni bat virus L (Shimoni)	ADD84511	457...1226	531...1343	25.0/ 43.3	1.00E-37
Rabies virus L (P18)	BAL22336	455...1216	529...1333	24.9/ 43.7	3.00E-37
Rabies virus L (BR-AL1)	BAK53488	455...1216	530...1334	24.8/ 43.9	4.00E-37
Rabies virus L (9001FRA)	ABZ81186	455...1216	529...1333	24.8/ 43.7	4.00E-37
Lagos bat virus L (8619NGA)	ABZ81171	455...1226	529...1343	25.2/ 42.6	5.00E-37
Rabies virus L (Coati-3639)	AFN24349	455...1216	529...1333	25.1/ 43.6	5.00E-37
Rabies virus L (BR-DR1)	BAK53493	455...1216	530...1334	24.8/ 43.7	5.00E-37
Rabies virus L (3634DR)	AFN24469	455...1216	529...1333	24.8/ 43.7	6.00E-37
Rabies virus L (WA173)	AEZ55970	455...1216	530...1334	24.9/ 43.6	6.00E-37
Rabies virus L (WA0173)	AFN24219	455...1216	529...1333	24.9/ 43.6	7.00E-37
Rabies virus L (ID7261)	AEZ55958	455...1216	530...1334	24.9/ 43.7	7.00E-37
Rabies virus L (FL385)	AFN24134	455...1216	529...1333	25.1/ 43.3	9.00E-37
Rabies virus L (Fl148)	AEZ55938	455...1216	268...1072	25.1/ 43.3	9.00E-37

pBLAST analysis was performed using the amino acid sequence of efEBLL-1 as a query. The non-redundant protein sequences (nr) was selected as the database. Top 50 hits are listed. [a] Strain names are not registered.

Supplementary table 2. EBLL elements in bat species

Species	Accession number	Contig	Region		*E*-value	Definition
			Contig (nt)	efEBLL-1 (aa)		
Eptesicus fuscus	ALEH01013293	contig013293	16050..21203	1..1718	0	efEBLL-1
	ALEH01077861	contig077861	19974..21296	1036..1476	7.00E-178	
			21305..21817	1484..1654	7.00E-178	
	ALEH01059268	contig059268	12988..12833	61..114	4.00E-04	
			12827..12702	116..157	4.00E-04	
			12705..12055	246..464	3.00E-10	
			12479..12420	324..343	3.00E-81	
			12400..11849	349..533	3.00E-81	
			11849..11430	534..673	3.00E-81	
			11222..11124	666..698	0.88	
			10451..10284	699..754	7.00E-08	
			9016..8777	1202..1281	5.00E-58	
			8726..7617	1299..1672	5.00E-58	
	ALEH01115181	contig115181	4117..3698	253..392	2.00E-49	
			4547..4101	96..255	2.00E-49	
			3692..3615	396..421	2.00E-49	
	ALEH01029091	contig029091	21221..21388	57..112	7.90E+00	
			21513..21722	779..848	5.00E-28	
			21763..21945	865..925	5.00E-28	
			21722..21766	849..863	5.00E-28	
			22000..22581	1367..1563	6.00E-42	
			22595..22900	1569..1671	6.00E-42	
Myotis davidii	ALWT01131278	contig131278	3393..4262	136..429	9.00E-148	
			4256..4798	428..608	9.00E-148	
			4795..4878	608..635	9.00E-148	
			4887..5234	639..754	9.00E-148	
			6304..6534	74..150	3.00E-16	
			7069..7356	712..807	9.00E-06	
			7362..7406	810..824	9.00E-06	
			8469..8843	820..944	5.00E-36	
			8816..10150	934..1379	2.00E-176	
			10159..10647	1382..1546	2.00E-176	
			12839..12976	1..46	3.00E-04	
	ALWT01141698	contig141698	3805..3734	974..997	2.00E-135	
			3729..3040	999..1228	2.00E-135	

Supplementary table 2. EBLL elements in bat species (continued)

Species	Accession number	Contig	Region		*E*-value	Definition
			Contig (nt)	efEBLL-1 (aa)		
Myotis davidii	ALWT01141698	contig141698	3046..2420	1226..1435	2.00E-135	
			1899..1528	1470..1595	6.00E-38	
			1534..1163	1595..1718	6.00E-38	
	AAPE02049592	cont2.49591	43612..43319	1..98	2.00E-25	
			43322..43200	98..138	2.00E-25	
	ALWT01026930	contig26930	18095..17574	485..662	5.00E-15	
			17943..17485	536..689	3.00E-21	
			17471..17322	903..952	5.00E-04	
			16929..16795	972..1016	7.00E-101	
			16735..16016	1042..1281	7.00E-101	
			15965..14919	1299..1659	7.00E-101	
	ALWT01174464	contig174464	2422..2276	776..824	8.00E-100	
			2271..1924	826..942	8.00E-100	
			1924..1754	943..1000	8.00E-100	
			1772..1179	999..1200	8.00E-100	
			1392..802	1127..1324	3.00E-14	
			841..176	1313..1490	2.00E-26	
			170..33	1493..1540	2.00E-26	
	ALWT01055717	contig55717	6431..6282	903..952	1.00E-22	
			6276..6196	957..983	1.00E-22	
			6172..6098	1104..1128	1.00E-29	
			6096..5797	1130..1232	1.00E-29	
			5592..5167	1237..1378	1.00E-85	
			5152..4409	1384..1629	1.00E-85	
			4473..4147	1610..1718	3.00E-06	
	ALWT01213390	contig213390	1741..2319	313..505	7.00E-71	
			2336..2470	513..557	7.00E-71	
			4334..4942	1060..1263	4.00E-78	
			4935..5210	1264..1356	4.00E-78	
			5233..5586	1601..1718	0.002	
	ALWT01131279	contig131279	1..267	49..137	6.00E-12	
			821..1099	1131..1223	1.00E-60	
			1092..1526	1221..1369	1.00E-60	
	ALWT01291348	contig291348	672..421	928..1015	2.00E-08	
			592..83	957..1128	1.00E-55	

Supplementary table 2. EBLL elements in bat species (continued)

Species	Accession number	Contig	Region		*E*-value	Definition
			Contig (nt)	efEBLL-1 (aa)		
Myotis davidii	ALWT01291348	contig291348	81..1	1130..1156	1.00E-55	
	ALWT01042510	contig42510	6083..6892	70..340	4.00E-50	
	ALWT01098736	contig98736	1649..1741	156..186	3.00E-36	mdEBLL-5
			1795..2055	205..290	3.00E-36	
			2052..2126	288..312	3.00E-36	
			2147..2536	321..455	3.00E-36	
			2635..2679	490..504	3.00E-36	
			2694..2882	511..574	3.00E-36	
			3309..3473	582..638	1.00E-06	
	ALWT01026931	contig26931	852..691	61..114	6.00E-08	
			701..534	112..167	6.00E-08	
			575..327	244..327	6.00E-36	
			360..289	318..341	3.70E+00	
			271..2	346..436	6.00E-36	
	ALWT01215306	contig215306	30350..30204	1..49	5.00E-21	
			30202..29924	52..145	5.00E-21	
Myotis lucifugus	AAPE02049592	cont2.49591	43612..43319	1..98	2.00E-25	mlEBLL-2,3,
			43322..43200	98..138	2.00E-25	
			39727..39509	136..208	5.00E-78	
			39506..38889	210..415	5.00E-78	
			38905..38567	403..524	4.00E-82	
			38567..38319	524..606	4.00E-82	
			38316..37672	608..823	4.00E-82	
			36537..36385	844..894	4.00E-11	
			36344..36027	281..400	8.00E-28	
			35866..35210	440..657	8.00E-26	
			35612..34719	524..823	2.00E-34	
			33657..33379	820..912	0	
			33346..33029	914..1019	0	
			33018..32428	1024..1221	0	
			32432..32139	1221..1318	0	
			32097..31957	1333..1379	0	
			31948..31463	1382..1546	0	
			28940..28557	10..137	2.00E-25	
			26884..26789	796..827	5.20E+00	

Supplementary table 2. EBLL elements in bat species (continued)

Species	Accession number	Contig	Region		*E*-value	Definition
			Contig (nt)	efEBLL-1 (aa)		
Myotis lucifugus	AAPE02049592	cont2.49591	26573..26409	832..886	2.00E-122	
			26409..26305	894..928	2.00E-122	
			26314..25463	925..1213	2.00E-122	
			25014..24568	1221..1369	2.00E-25	
			10099..9508	1134..1223	2.00E-49	
			9606..9402	1332..1366	2.00E-49	
	AAPE02025596	cont2.25595	555..2795	310..1059	0	mlEBLL-1
			6266..6499	1059..1137	7.00E-167	
			6510..7442	1142..1452	7.00E-167	
			7417..7791	1445..1569	7.00E-167	
			7833..8246	1581..1718	7.00E-167	
	AAPE02006259	cont2.6258	26414..26620	903..973	4.00E-111	mlEBLL-5
			26625..27077	976..1126	4.00E-111	
			27077..27199	1130..1170	4.00E-111	
			27193..27360	1169..1225	4.00E-111	
			27590..27727	1237..1278	2.00E-100	
			27786..28028	1299..1379	2.00E-100	
			28040..28606	1384..1573	2.00E-100	
			28605..29036	1575..1718	2.00E-100	
	AAPE02020529	cont2.20528	3683..3498	628..689	3.00E-09	mlEBLL-7
			3510..3310	895..958	4.00E-09	
			2944..2714	972..1048	2.00E-18	
			2748..1432	1042..1528	3.00E-58	
			1676..1398	1440..1537	1.00E-13	
			1297..1046	1573..1658	1.00E-13	
	AAPE02026110	cont2.26109	118007..117591	1051..1190	7.00E-52	
			117505..117191	1220..1324	7.00E-52	
			117230..117027	1313..1379	2.00E-30	
			117015..116320	1384..1617	2.00E-30	
			116689..116465	1493..1568	0.058	
	AAPE02024702	cont2.24701	25452..25291	135..186	7.00E-40	mlEBLL-6
			25237..24977	205..290	7.00E-40	
			24980..24495	288..455	7.00E-40	
			24433..24128	477..581	7.00E-40	
			23706..23542	582..638	2.00E-06	

Supplementary table 2. EBLL elements in bat species (continued)

Species	Accession number	Contig	Region		*E*-value	Definition
			Contig (nt)	efEBLL-1 (aa)		
Myotis lucifugus	AAPE02001885	cont2.1884	6473..5916	230..416	6.00E-38	
	AAPE02066775	cont2.66774	6541..6870	1470..1586	6.00E-31	
			6914..7252	1606..1718	6.00E-31	
	AAPE02023767	cont2.23766	35168..34863	1478..1586	3.00E-29	
			34819..34481	1606..1718	3.00E-29	

tBLASTn search was conducted using the amino acid sequence of efEBLL-1 as a query. The whole genome shotgun sequences (WGS) of *Epteticus fuscus*, *Myotis davidii*, and *Myotis lucifugus* were selected as the database. Sequences hit with the e-value < -20 were considered to be homologous to efEBLL-1.

Supplementary Table 3. Primers used for PCR in this study

Name	Sequence	Orientation	Note
MH185	GTGTCATGCTCATATGGAAAGCAGTTG	Forward	Detection of EBLL-1s
MH186	GTAGACTCTTGATCGGTATGTGTCAGC	Reverse	Detection of EBLL-1s
MH187	CATAGAGCTGATGAAAGAGGGGTTCAG	Forward	Detection of EBLL-1s
MH188	AAAACAATCCGAGCCAAACATTCTGTC	Reverse	Detection of EBLL-1s
MH189	AGCAAAACTCCAAATTCCCAATGAACC	Forward	Detection of EBLL-1s
MH190	TGTAGCAGTCTCCTGAAGGTGAAATTG	Reverse	Detection of EBLL-1s
MH191	CATGGTACCGGGCCAAGACCTCTTGGAATTTATATG	Forward	Cloning of EBLL-1s
MH192	CATGGGCCCAATCATATTACCATGTCACGGTAGGGC	Reverse	Cloning of EBLL-1s
MH217	CAGCCACAGGAGGAAATATACTACAGC	Forward	Sequencing of the upstream of EBLL-1s
MH218	GAGTTCTCGAACATTTGGGAAGCATTG	Reverse	Sequencing of the upstream of EBLL-1s
MH221	GGATGTCAAATGCATACACAAGTCCAC	Forward	Sequencing of the downstream of EBLL-1s
MH222	ATATGCAGCTCATACACACGAAACAAC	Reverse	Sequencing of the downstream of EBLL-1s
MH236	CCCCACTGGAGTTTTCCATTTTGATG	Forward	Sequencing of RAG2
MH237	AATTTTCCCAGAACCCTTTTTGTGGAG	Reverse	Sequencing of RAG2

Underlined letters indicate restriction sites used for the cloning.

Supplementary Table 4. Domain families in bornavirus L proteins and EBLL-1s

Name	Length (aa)	Region	Domain	Pfam Accession	Pfam *E*-value
ABV L	1712	143...956	Mononegavirales RNA dependent RNA polymerase	PF00946	1.90E-174
		994...1221	Mononegavirales mRNA-capping region V	PF14318	6.40E-46
BDV L	1711	45...956	Mononegavirales RNA dependent RNA polymerase	PF00946	1.20E-200
		993...1220	Mononegavirales mRNA-capping region V	PF14318	7.70E-43
efEBLL-1	1718	129...948	Mononegavirales RNA dependent RNA polymerase	PF00946	2.50E-124
		985...1209	Mononegavirales mRNA-capping region V	PF14318	1.90E-33
enEBLL-1	1718	131...948	Mononegavirales RNA dependent RNA polymerase	PF00946	5.10E-125
		985...1209	Mononegavirales mRNA-capping region V	PF14318	7.00E-33
esEBLL-1	1718	129...948	Mononegavirales RNA dependent RNA polymerase	PF00946	5.20E-125
		985...1209	Mononegavirales mRNA-capping region V	PF14318	6.50E-33

Domains were searched by Pfam using the amino acid sequences of the ABV L, BDV L, efEBLL-1, esEBLL-1 or enEBLL-1.
